# Isotopic constraints confirm the significant role of microbial nitrogen oxides emissions from the land and ocean environment

**DOI:** 10.1093/nsr/nwac106

**Published:** 2022-06-03

**Authors:** Wei Song, Xue-Yan Liu, Benjamin Z Houlton, Cong-Qiang Liu

**Affiliations:** School of Earth System Science, Tianjin University, Tianjin 300072, China; School of Earth System Science, Tianjin University, Tianjin 300072, China; Department of Global Development and Department of Ecology and Evolutionary Biology, Cornell University, Ithaca, NY 14850, USA; School of Earth System Science, Tianjin University, Tianjin 300072, China

**Keywords:** nitrogen isotopes, nitrate, NO_x_ emission, nitrogen deposition, microbial N cycle

## Abstract

Nitrogen oxides (NO_x_, the sum of nitric oxide (NO) and N dioxide (NO_2_)) emissions and deposition have increased markedly over the past several decades, resulting in many adverse outcomes in both terrestrial and oceanic environments. However, because the microbial NO_x_ emissions have been substantially underestimated on the land and unconstrained in the ocean, the global microbial NO_x_ emissions and their importance relative to the known fossil-fuel NO_x_ emissions remain unclear. Here we complied data on stable N isotopes of nitrate in atmospheric particulates over the land and ocean to ground-truth estimates of NO_x_ emissions worldwide. By considering the N isotope effect of NO_x_ transformations to particulate nitrate combined with dominant NO_x_ emissions in the land (coal combustion, oil combustion, biomass burning and microbial N cycle) and ocean (oil combustion, microbial N cycle), we demonstrated that microbial NO_x_ emissions account for 24 ± 4%, 58 ± 3% and 31 ± 12% in the land, ocean and global environment, respectively. Corresponding amounts of microbial NO_x_ emissions in the land (13.6 ± 4.7 Tg N yr^−1^), ocean (8.8 ± 1.5 Tg N yr^−1^) and globe (22.5 ± 4.7 Tg N yr^−1^) are about 0.5, 1.4 and 0.6 times on average those of fossil-fuel NO_x_ emissions in these sectors. Our findings provide empirical constraints on model predictions, revealing significant contributions of the microbial N cycle to regional NO_x_ emissions into the atmospheric system, which is critical information for mitigating strategies, budgeting N deposition and evaluating the effects of atmospheric NO_x_ loading on the world.

## Teaser

This study constructed new isotope mass-balance models, constrained fractions and emission amounts of major nitrogen oxides sources in land and ocean systems, and confirmed the significant contribution of microbial nitrogen cycles to global nitrogen oxides emissions.

## INTRODUCTION

Atmospheric nitrogen oxides (NO_x_) loading influence human health (e.g. respiratory and cardiovascular diseases, acute bronchitis) [1], tropospheric chemistry (e.g. precipitation acidity, aerosol and ozone formation) [2–4], climate [4] and economic development [5]. In past decades, anthropogenic NO_x_ emissions have significantly increased the fluxes of atmospheric NO_3_^−^ deposition [6–8], altered N cycles in both terrestrial and marine ecosystems [9–12] and thus affected microbial NO_x_ emissions to the atmosphere [13]. Hence, it is pivotal to accurately constrain land and ocean NO_x_ emissions to the atmosphere to mitigate human-induced NO_x_ emissions, budget NO_3_^−^ deposition fluxes and evaluate the eco-environmental and climatic effects of atmospheric NO_x_ loading. However, it has long been challenging to accurately constrain land- and ocean-to-atmosphere NO_x_ emissions due to uncertainties over microbial N cycles in both land and ocean.

In marine environments, the oil combustion of marine traffic transportation is a known source of NO_x_ emissions [14–20]. According to the European Monitoring and Evaluation Programme Meteorological Synthesizing Centre West model, NO_x_ emissions from oil combustion in the ocean averaged 6.4 ± 0.8 Tg N yr^−1^ (5.0–7.8 Tg N yr^−1^) [14–20]. However, the microbial N cycle occurring in the ocean is the other significant source of NO_x_ emissions [21–24]. First, earlier studies based on molecular analysis and lab culture experiments have confirmed that multiple kinds of bacteria associated with several processes of microbial N cycles can produce NO, e.g. ammonium-oxidizing bacteria, nitrite-oxidizing bacteria, methanotrophic bacteria and denitrifying bacteria [25–29]. Second, nitrification in the oxic layer of the ocean is a significant source of NO [22] and NO can be produced in biofilms and marine sediments [30]. Third, *Ulva prolifera* (forming a belt on a vertical concrete wall in the upper intertidal zone at low tide) was the primary contributor to the high NO concentrations during the late-bloom period [31]. Meanwhile, the photolysis of NO_2_^−^ and NO_3_^−^ (in the surface water and on particles) or alkyl nitrates or dissolved organic matter may also be the sources of atmospheric NO in the ocean [32–35]. However, due to its high reactivity [36], NO would be involved quickly into the NO_x_ cycle in the atmosphere [34]. Accordingly, it has long been difficult to accurately observe microbial NO emissions in the ocean [24]. Until now, microbial NO_x_ emissions from the ocean and their fractional contribution to total NO_x_ emissions from the ocean have not been quantified [21–24]. Hitherto, owing to the lack of microbial NO_x_ emissions, the NO_x_ from oil combustion has long been assumed as the total ocean NO_x_ emissions in reports of the Intergovernmental Panel on Climate Change (IPCC) [20].

In the land environment, NO_x_ emissions are mainly derived from coal combustion, oil combustion, biomass burning and microbial N cycles in substrates such as waters, soils and wastes [3,37–40]. Currently, emission amounts of NO_x_ from coal combustion [10,41], oil combustion [42] and biomass burning [43,44] have been reported explicitly in national statistic yearbooks and emission inventories [45–47]. However, land NO_x_ emissions from microbial N cycles have been observed chiefly for soils under natural vegetation and agriculture [40,43,48]. Therefore, estimates of NO_x_ emissions from the land are based on limited empirical observations combined with process and statistical models and satellites used to scale up emissions [40,49,50]. Based on IPCC reports, microbial NO_x_ emissions were budgeted at 5.6 Tg N yr^−1^ before 2001, increasing to 11.0 Tg N yr^−1^ when incorporating more observational data in the report of 2013 [40,49,50]. This doubling of emissions highlights a substantial underestimation of microbial NO_x_ emissions in the land, which has shifted with additional measurements and better models. New methods are strongly needed to comprehensively constrain microbial NO_x_ emissions from soils and many other unconsidered substrates (such as the surface water of rivers, lakes, swamps, etc.) and emission sources (such as wastewater, water treatment systems, solid wastes).

**Figure 1.**
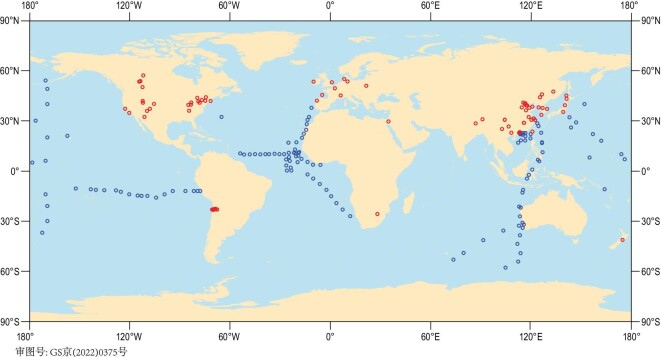
The distribution of study sites with δ^15^N_p-NO3-_ observations. Red and blue circles represent land sites (*n* = 91) and ocean sites (*n* = 134), respectively.

**Figure 2.**
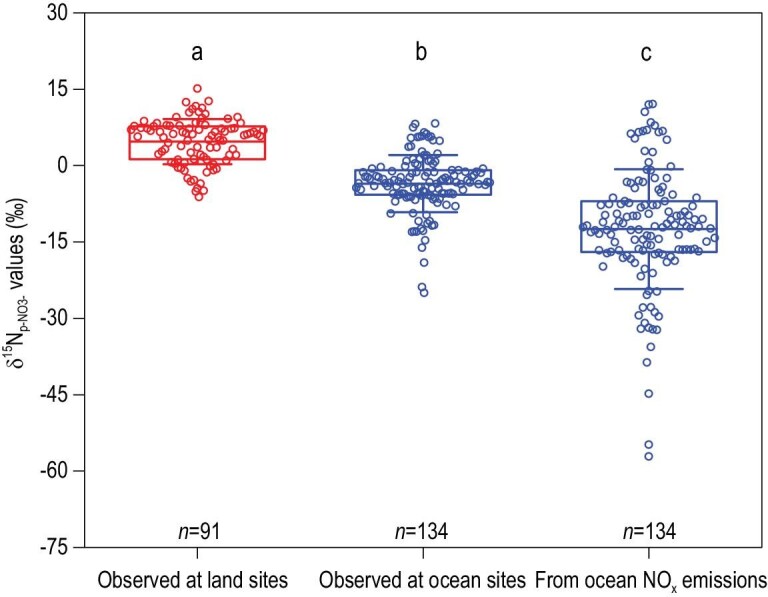
δ^15^N_p-NO3-_ values observed at land sites, observed at ocean sites and derived from ocean NO_x_ emissions. Circles around each box show mean values of replicate measurements at each site (*n*) (replicate measurements at each site are 1−318 and 1−72 for land and ocean sites, respectively). The box encompasses the 25^th^ to 75^th^ percentiles; whiskers and lines in boxes are the SD and mean values, respectively. Different letters above the boxes show significant differences at *P *< 0.05.

Here we provided a unique evaluation of the relative importance of the microbial NO_x_ emissions in the land and ocean to the known fossil-fuel NO_x_ emissions and then made a new budget for global microbial NO_x_ emissions. First, we compiled stable N isotopes (δ^15^N values) of NO_3_^−^ in atmospheric particulates (denoted as δ^15^N_p-NO3__-_ hereafter) in the land and ocean, respectively (detailed in ‘Materials and methods’ section) (Fig. 1 and Supplementary Table S1). Second, based on concentrations and δ^15^N of NO_x_, HNO_3_ and p-NO_3_^−^ over the land, we estimated the δ^15^N of the initial NO_x_ mixture from different emission sources in the atmosphere (denoted as δ^15^N_i-NOx_, Supplementary Fig. S1) and the difference between δ^15^N_p-NO3__-_ and δ^15^N_i-NOx_ values (denoted as ^15^Δ_i-NOx→p-NO3__-_) (detailed in ‘Materials and methods’ section). By using ^15^Δ_i-NOx→p-NO3__-_ (Supplementary Fig. S2), δ^15^N_p-NO3__-_ (Fig. 2) and δ^15^N of dominant sources of NO_x_ emissions (coal combustion, oil combustion, biomass burning and microbial N cycles, Supplementary Table S2), we estimated the relative contributions of dominant NO_x_ sources from the land and ocean, respectively, by developing a model of Stable Isotope Analysis in R code (detailed in ‘Materials and methods’ section). Finally, combining fractional contributions with corresponding amounts of fossil-fuel NO_x_ emissions from the land and the ocean, we calculated the amount of microbial NO_x_ emissions in the land and ocean, respectively (detailed in ‘Materials and methods’ section).

## RESULTS AND DISCUSSION

### Different δ^15^N signatures of atmospheric p-NO_3_^−^ between the land and ocean

Mean δ^15^N_p-NO3__-_ observed over terrestrial sites (4.7 ± 3.6‰; *n *= 91) was significantly higher (*p *< 0.05) than that observed for ocean sites (–3.5 ± 3.9‰; *n *= 134) (Fig. 2). This finding implied that human activities contributed relatively more ^15^N-enriched NO_x_ to atmospheric NO_x_ loading on the land than in the ocean.

First, the δ^15^N_p-NO3__-_ signal observed at land sites can represent land NO_x_ emissions without a significant overprinting of marine sources. The net water vapor flux transported from the ocean to the land accounted for only 10% of the total water evaporation over the ocean [51,52]. According to the existing oceanic NO_x_ emissions (6.4 ± 0.8 Tg N yr^−1^ based on the known oil combustion) [14–20] and the land NO_x_ emissions (53.3 ± 4.6 Tg N yr^−1^) [43,53–58], the ocean-to-land atmospheric transport of NO_x_ accounts for only 1.2% of land NO_x_ emissions and thus is often assumed negligible [35]. Accordingly, the δ^15^N_p-NO3__-_ values observed at land sites can be directly used to differentiate dominant sources of NO_x_ emissions (Equation 5 in the online Supplementary Data).

However, the δ^15^N_p-NO3-_ signal observed at ocean sites cannot represent the NO_3_^−^ purely derived from ocean NO_x_ emissions. Because the land has much higher NO_x_ emissions and a smaller area, and thus a higher concentration than the ocean [57,59,60], the net transportation of atmospheric NO_x_ occurs from the land to the ocean. The modeled NO_y_ (the sum of NO_x_, inorganic and organic nitrates in the atmosphere) transportation (11.0 Tg N yr^−1^) [61] is about 1.7 times the oceanic and accounts for 21% of land NO_x_ emissions. Accordingly, the δ^15^N signals of p-NO_3_^−^ derived from the land-to-ocean NO_y_ transportation should be excluded (Equation 2 in the online Supplementary Data) to obtain the δ^15^N values of p-NO_3_^−^ derived only from the ocean NO_x_ emissions (Supplementary Fig. S1) to differentiate the relative contributions between oil combustion and microbial NO_x_ emissions (Equation 6 in the online Supplementary Data). Besides, the land-derived NO_x_ and p-NO_3_^−^ are the dominant form of the land-to-ocean NO_y_ transportation and between them, the p-NO_3_^−^ is the main type to be transported because the lifetime of NO_x_ is much shorter [35,61,62]. So far, no substantial isotope effect was assumed for the physical processes of atmospheric transportation [63,64]. Thus, we thought that the ocean p-NO_3_^−^ produced by the land-derived NO_x_ did not differ isotopically from the land p-NO_3_^−^ and used isotope mass-balance calculations to obtain the δ^15^N values of p-NO_3_^−^ derived only from the ocean NO_x_ emissions (Equation 2 in the online Supplementary Data).

The calculated results revealed that the δ^15^N of p-NO_3_^−^ purely derived from ocean NO_x_ emissions averaged –12.5 ± 8.2‰ (Fig. 2), which was much lower than the δ^15^N_p-NO3-_ observed for the land sites (4.7 ± 3.6‰; Fig. 2). The increase in ^15 ^N/^14^N of p-NO_3_^−^ over the land should be mainly influenced by ^15^N-enriched NO_x_ sourced to coal combustion, which was distinctly elevated in δ^15^N values (mean = 14.2 ± 5.1‰, Supplementary Table S2). However, the lower ^15 ^N/^14^N of p-NO_3_^−^ derived from ocean NO_x_ emissions revealed a microbial NO_x_ source with distinctly lower δ^15^N values than other sources (mean = −37.0 ± 13.5‰, Supplementary Table S2). Our findings demonstrated the contrasting δ^15^N pattern between p-NO_3_^−^ derived from the land and ocean NO_x_ emissions. Moreover, the newly constrained δ^15^N of p-NO_3_^−^ sourced to ocean NO_x_ emissions provided a more accurate and straightforward opportunity to constrain source contributions and emission amounts via isotope modeling.

### Relative contributions of dominant NO_x_ sources to p-NO_3_^−^

δ^15^N_p-NO3-_ values are determined by the δ^15^N of sources and their relative contributions to total NO_x_ emission and isotope effects of the NO_x_ transformation to p-NO_3_^−^ (^15^Δ_i-NOx→p-NO3-_ values) [65]. Accordingly, we compiled δ^15^N values of dominant sources of NO_x_ emissions (Supplementary Table S2), constrained ^15^Δ_i-NOx→p-NO3-_ values (Supplementary Fig. S2) and thereby constructed isotope mass-balance models to further evaluate the contribution of dominant NO_x_ sources to p-NO_3_^−^ in the land and ocean, respectively (detailed in ‘Materials and methods’ section).

**Figure 3.**
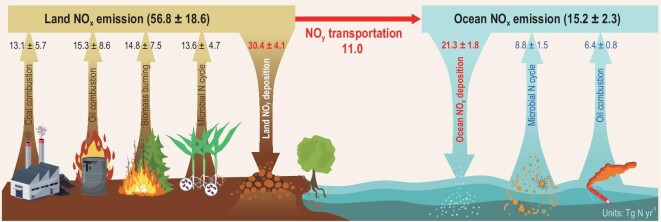
Emissions of significant land and ocean NO_x_ sources (in black and blue) based on natural isotope methods (detailed in ‘Materials and methods’ section). Data of the NO_y_ deposition and transportation (in red) were cited from Refs [35,61,62,70,89,90].

For source δ^15^N end-members, we considered coal combustion, oil combustion, biomass burning and the microbial N cycle as dominant NO_x_ sources of p-NO_3_^−^ over the land [65], while oil combustion and the microbial N cycle are dominant NO_x_ sources to p-NO_3_^−^ over the ocean [2,20]. The δ^15^N of such sources differ significantly from each other (*p *< 0.05, Supplementary Table S2), which is a prerequisite to differentiating their relative contributions isotopically. We assumed the same δ^15^N value of each NO_x_ source for both land and ocean sites due to no δ^15^N observations on NO_x_ from oil combustion and microbial N cycle in the ocean (detailed in ‘Materials and methods’ section). We did not consider lightning a dominant NO_x_ source because the NO_x_ produced by lightning in the land and ocean atmosphere is negligible. First, the global NO_x_ production from lighting is 5.2 ± 1.0 Tg N yr^−1^ (Supplementary Text S1), which accounted for ∼9.7% and ∼7.2% of global NO_x_ emissions by modeling methods (51.9–58.0 Tg N yr^−1^) and by isotopic methods in this study (Fig. 3). Moreover, the meridional distribution of global lightning in the atmosphere shows three main lightning centers of the Americas, Africa and the maritime continent in Southeast Asia. The minima represent the oceanic regions where little lightning is observed [66]. This baseline assumption of the dominant NO_x_ sources is supported by emission inventory and deposition modeling [10,41,42,45–47].

Regarding isotope effects, we estimated ^15^Δ_i-NOx→p-NO3-_ values under two independent scenarios (detailed in ‘Materials and methods’ section) and found no significant differences between them (11.3 ± 2.1‰ and 13.1 ± 3.8‰, respectively) (Supplementary Fig. S2). Accordingly, we used the mean ^15^Δ_i-NOx→p-NO3-_ estimate (12.2 ± 2.2‰) in our subsequent isotope mass-balance calculations (Supplementary Fig. S2). The mean ^15^Δ_i-NOx→p-NO3-_ value in this study (12.2 ± 2.2‰) did not differ from the ϵ_NO→p-NO3-_ value estimated by Li *et al.* [67] (∼15‰) and was also comparable with the global mean ^15^Δ_i-NOx→p-NO3-_ value (16.7 ± 2.3‰) [65]. The calculation of the global mean ^15^Δ_i-NOx→p-NO3-_ value by Song *et al.* [65] was based on the theoretical framework of computation established by Walters and Michalski [68,69], which combined natural ^15^N and ^17^O isotopes with environmental parameters relating to the NO_x_ oxidization to p-NO_3_^−^. Relative contributions of dominant NO_x_ sources were calculated using the Stable Isotope Analysis model in R programming language (detailed in ‘Materials and methods’ section). Results showed that the NO_x_ from coal combustion, oil combustion, biomass burning and microbial N cycle accounted for 23 ± 7%, 27 ± 11%, 26 ± 10% and 24 ± 4% on the land, respectively (Supplementary Fig. S3a). In contrast, the NO_x_ from oil combustion and microbial N cycle accounted for 42 ± 3% and 58 ± 3% in the ocean, respectively (Supplementary Fig. S3a). Generally, high fractions of microbial NO_x_ emissions revealed the vital contribution of this pathway to both land and ocean NO_x_ emissions into the global atmosphere.

### Total and microbial NO_x_ emissions on the land

Based on statistical data on quantities and NO_x_ emission factors of coal and oil combustions in the land system, previous studies have estimated global fossil-fuel NO_x_ emissions with a relatively high degree of certainty [7,43,50,70,71]. Global fossil-fuel NO_x_ emissions averaged 28.4 ± 1.8 Tg N yr^−1^, showing a relatively low variation over past decades (25.6–30.0 Tg N yr^−1^) [7,43,50,70,71]. By using the fraction and amount of fossil-fuel NO_x_ emissions in the land (50 ± 14% and 28.4 ± 1.8 Tg N yr^−1^, respectively, Supplementary Fig. S3a), we estimated that total land NO_x_ emissions were 56.8 ± 18.6 Tg N yr^−1^ (Fig. 3 and Supplementary Fig. S3b). Our estimate falls in the range of the total land NO_x_ emissions (50.0–61.4 Tg N yr^−1^; averaging 55.6 ± 2.9 Tg N yr^−1^) estimated by optimized modeling methods by considering more microbial sources of NO_x_ emissions [54,57,58]. However, our estimate is higher than the total land NO_x_ emissions (39.7–51.0 Tg N yr^−1^; averaging 43.8 ± 5.0 Tg N yr^−1^) estimated using the global NO_2_ satellite column concentrations [43,55,56]. Due to no consideration of the influences of atmospheric NO_2_ transformations, the estimates based on the satellite data were thought to underestimate global NO_x_ emissions [72–74].

Based on the fraction and amount of total land NO_x_ emissions (24 ± 4% and 56.8 ± 18.6 Tg N yr^−1^, respectively, Fig. 3 and Supplementary Fig. S3), microbial NO_x_ emissions on the land were calculated as 13.6 ± 4.7 Tg N yr^−1^ (Fig. 3 and Supplementary Fig. S3b). So far, observations on microbial NO_x_ emissions on the land showed a relatively lower flux of 7.9 ± 1.5 Tg N yr^−1^ (5.0–11.0 Tg N yr^−1^; data compiled from Refs [43,55,75–84]) than our estimate, because these observations have been conducted mainly on fertilized soils and merely on unfertilized soils and other land substrates. Besides, few modeling studies showed distinctly higher fluxes of land microbial NO_x_ emissions ≤20.4 Tg N yr^−1^ [80] and 23.6 Tg N yr^−1^ [85] than the observation results and our estimate, due to overestimated N inputs in cropland and natural ecosystems and largely overlooked the influence of NO_x_ sink uncertainties on the satellite-derived NO_x_ fluxes. However, by considering more substrates of microbial N cycles on the land to optimize the modeling methods, some studies showed the land microbial NO_x_ emissions as 11.5–13.6 Tg N yr^−1^ (12.4 ± 0.7 Tg N yr^−1^) [53,71,86,87], which is very comparable with our estimate. The isotopic method in our study offers a comprehensive and accurate constraining on microbial NO_x_ emissions.

### Total and microbial NO_x_ emissions in the ocean

Based on statistical data of quantities and NO_x_ emission factors of oil combustions in the ocean system, ocean fossil-fuel NO_x_ emissions have been estimated as 6.4 ± 0.8 Tg N yr^−1^ on average (5.0–7.8 Tg N yr^−1^; compiled from [14–20]). Using the fraction of the ocean fossil-fuel NO_x_ emissions in our study (42 ± 3%, Supplementary Fig. S3a), we estimated the total ocean NO_x_ emissions as 15.2 ± 2.3 Tg N yr^−1^ (Fig. 3 and Supplementary Fig. S3b). The ocean NO_y_ deposition averaged 21.3 ± 1.8 Tg N yr^−1^ (18.0–23.0 Tg N yr^−1^; compiled from Refs [35,61,62,88–90]), which includes the land-to-ocean NO_y_ transportation of 11.0 Tg N yr^−1^ [61]. Accordingly, the oceanic NO_y_ deposition derived from oceanic NO_x_ emissions was 10.3 ± 1.8 Tg N yr^−1^, which is lower than our study's total ocean NO_x_ emissions. The generally higher NO_x_ emissions than NO_y_ deposition in the ocean might be attributed to other fates such as biological NO_x_ uptake and atmosphere retention. Further, we calculated ocean microbial NO_x_ emissions as 8.8 ± 1.5 Tg N yr^−1^ on average (Fig. 3 and Supplementary Fig. S3b). Our results updated the total and microbial NO_x_ emissions in the marine environment.

### Total and microbial NO_x_ emissions in the globe

By integrating the land and ocean values together (detailed in ‘Materials and methods’ section), we calculated global total NO_x_ emissions as 72.0 ± 18.1 Tg N yr^−1^ (Fig. 3 and Supplementary Fig. S3b). Before this work, the modeled total land NO_x_ emissions (39.7–61.4 Tg N yr^−1^; compiled from Refs [43,53–58]) have been assumed as the global NO_x_ emissions because the ocean NO_x_ emissions have been unconstrained. Our results showed that oceanic NO_x_ emissions accounted for ∼21% of the global NO_x_ emissions. The global NO_x_ emissions have been underestimated by 15–45% because oceanic NO_x_ emissions have been unconsidered.

Moreover, we found that microbial NO_x_ emissions accounted for 31 ± 12% of the total NO_x_ emissions globally and reached up to 22.5 ± 4.7 Tg N yr^−1^ (Fig. 3 and Supplementary Fig. S3b). By comparison, microbial NO_x_ emissions in the land (13.6 ± 4.7 Tg N yr^−1^), ocean (8.8 ± 1.5 Tg N yr^−1^) and globe (22.5 ± 4.7 Tg N yr^−1^) are ∼0.5, 1.4 and 0.6 times fossil-fuel NO_x_ emissions in the land, ocean and globe, respectively (Fig. 3 and Supplementary Fig. S3b). Our results highlight a vital role of the microbial N cycle in global NO_x_ emissions. In addition to the direct impacts of fossil-fuel combustion on global NO_x_ emissions, other human activities such as inefficient fertilizer use in cropping systems, wastes and sewage discharge and treatments, N deposition and water N enrichment all can accelerate microbial NO_x_ emissions in the land, inland water bodies, estuaries and ocean [13,91].

Our results offer an updated and isotopically grounded estimate of land- and ocean-to-atmosphere NO_x_ emissions. Notably, our results revealed that previous reports have largely underestimated land-based microbial NO_x_ emissions, constrained long-missing uncertainties over ocean microbial NO_x_ emissions and therefore elevated the recognition of the substantial contribution of the microbial N cycle to global NO_x_ emissions. Moreover, our findings highlight the unique significance of natural records of atmospheric N isotopes for understanding global N biogeochemical cycles. Currently, reducing NO_x_ emissions to alleviate N pollution while sustaining economic development is a major challenge in the twenty-first century**.** Owing partly to unclear contributions of microbial processes to NO_x_ emissions, many countries have been engaging in developing technologies and measures for reducing fossil-fuel NO_x_ emissions to reduce airborne and water N pollution, with a focus on adjusting energy systems and increasing the chemical conversion of NO_x_ to reduce emissions during fossil-fuel combustion. Our findings point to the need to consider the substantial contribution of the microbial N cycle to atmospheric NO_x_ loadings while reducing fossil-fuel NO_x_ emissions. Accordingly, the potential costs and impacts of reducing fossil-fuel NO_x_ emissions need to be re-assessed when making more effective emission mitigation strategies—including the indirect effects of anthropogenic N on terrestrial and marine microbial processes. Moreover, the isotopically constrained microbial NO_x_ emissions and updated total NO_x_ emissions we provide are helpful for benchmarking atmospheric and earth system models that project the feedback between the biosphere, climate and global N cycle.

In summary, based on large-scale isotope observations of p-NO_3_^−^ in the atmosphere, we established a simple but effective approach for estimating NO_x_ sources in the atmosphere. Before, isotope mass-balance models have been constructed to successfully partition continental hydrologic fluxes and quantify the contributions of local evaporation and ocean-to-land water transportation to the land moisture [92,93]. Accordingly, the framework established in our study enriches the application of isotopic mass-balance approaches in quantifying processes and fluxes of global biogeochemical cycles. However, our method can only consider dominant sources of NO_x_ emissions. Additional work on detailed measurements of *δ*^15^N values for all NO_x_ emission sources could further refine our estimates. Isotope observations of p-NO_3_^−^ in the atmosphere across more sampling areas will be critical to reducing uncertainties in our estimation and offering spatial tools to pinpoint source regions of great concern.

## MATERIALS AND METHODS

Detailed materials and methods are given in the online supplementary materials.

## Supplementary Material

nwac106_Supplemental_File
