# Supplementary material for: Isotopic constraints confirm the significant role of microbial nitrogen oxides emissions from the land and ocean environment
Source: Natl Sci Rev. 2022 Jun 3;9(9):nwac106. doi: 10.1093/nsr/nwac106 (PMC9477198; doi:10.1093/nsr/nwac106)
Supplement: nwac106_Supplemental_File [file nwac106_supplemental_file.pdf]

Supplementary Materials for

Isotopic constraints confirm the significant role of microbial nitrogen oxides emissions from the land and ocean environment

Wei Song[1], Xue-Yan Liu[1*], Benjamin Z. Houlton[2], Cong-Qiang Liu[1]

[1]School of Earth System Science, Tianjin University, Tianjin, 300072, China;

[2]Department of Global Development and Department of Ecology and Evolutionary Biology, Cornell University, Ithaca, NY 14850, USA

*Corresponding author: Xue-Yan Liu

Email: liuxueyan@tju.edu.cn

This PDF file includes:

Supplementary Text: Materials and Methods

Supplementary Figures: Figs. S1 to S9

Supplementary Tables: Tables S1 to S3

SI References: 1-71

Supplementary Text: Texts S1 to S8

Supplementary Text

Materials and Methods

Global $\delta^{15}N_{p\text{-}NO3\text{-}}$ observations

Publications with $\delta^{15}N_{p\text{-}NO3\text{-}}$ observations were searched by using the keywords of 'nitrogen isotope', 'nitrate', 'particulate NO_3^-', 'dry deposition', and 'PM' in the databases of the Web of Science (1), Google Scholar (2), and Baidu Scholar (3). By August of 2021, we obtained 74 publications (listed in Text S2 and Table S1), which spanned the sampling years of 1974–2019 (Fig. S4). There were 4767 measurements of $\delta^{15}N_{p\text{-}NO3\text{-}}$ (Fig. S5) and 225 sampling sites when counting $\delta^{15}N_{p\text{-}NO3\text{-}}$ observations by different sites, i.e., observations in different sampling years at the same site were counted only as one site (Fig. 1). In this way, there were 91 land sites and 134 ocean sites (Fig. 1), in which land sites are mainly distributed in urban, rural, mountain, forest, and wetland areas. All sampling sites are distributed between 60° N and 60° S (Fig. 1). Due to the difficulty in collecting atmospheric NO_3^- in the ocean environment, the number of individual $\delta^{15}N_{p\text{-}NO3\text{-}}$ observations on the land (4424 measurements) is much more than over the ocean (343 measurements). To reduce the uncertainty caused by different individual $\delta^{15}N_{p\text{-}NO3\text{-}}$ observations between land and ocean, we used site-based mean $\delta^{15}N_{p\text{-}NO3\text{-}}$ values rather than individual observations for both land (91 sites) and ocean (134 sites) (Fig. 1). The 134 ocean sites have covered the Pacific Ocean, the Atlantic Ocean, and the Indian Ocean (Fig. 1). Until now, land $\delta^{15}N_{p\text{-}NO3\text{-}}$ observations have been conducted in East Asia, Europe, and North America, with very few in Africa, Russia, and South America (Fig. 1). Data in the figures were extracted using the software of Web Plot Digitizer (Version 4.2, San Francisco, California, USA) (4).

$\delta^{15}N$ values of p-NO_3^- derived from ocean NO_x emissions

The p-NO_3^- observed in the ocean is composed of p-NO_3^- derived from ocean NO_x (the sum of NO and NO_2) emissions and the p-NO_3^- transported from the land atmosphere (Eq. 1) (5). Accordingly, $\delta^{15}N_{p\text{-}NO3\text{-}}$ observed at ocean sites (denoted as $\delta^{15}N_{p\text{-}NO3\text{-}O}$) were determined by fractional contributions and $\delta^{15}N$ values of p-NO_3^-

derived from ocean NO_x emissions (f_E and $\delta^{15}N_{p\text{-}NO3\text{-}E}$, respectively) and $p\text{-}NO_3^-$ transported from the land atmosphere (denoted as f_T and $\delta^{15}N_{p\text{-}NO3\text{-}T}$, respectively).

$$\delta^{15}N_{p\text{-}NO3\text{-}O} = \delta^{15}N_{p\text{-}NO3\text{-}E} \times f_E + \delta^{15}N_{p\text{-}NO3\text{-}T} \times f_T \qquad \text{(Eq. 1)}$$

in which $f_E + f_T = 1$. Thus, Eq. 1 can be re-arranged to Eq. 2.

$$\delta^{15}N_{p\text{-}NO3\text{-}E} = (\delta^{15}N_{p\text{-}NO3\text{-}O} - \delta^{15}N_{p\text{-}NO3\text{-}T} \times f_T) / (1 - f_T) \qquad \text{(Eq. 2)}$$

In our calculations, the $\delta^{15}N_{p\text{-}NO3\text{-}O}$ is the site-based mean $\delta^{15}N_{p\text{-}NO3\text{-}}$ value at each ocean observation site (Figs. 1 & 2). The $\delta^{15}N_{p\text{-}NO3\text{-}T}$ is the mean value of site-based mean $\delta^{15}N_{p\text{-}NO3\text{-}}$ values, assuming that the $\delta^{15}N$ of $p\text{-}NO_3^-$ produced in the land atmosphere (i.e., the $\delta^{15}N_{p\text{-}NO3\text{-}}$ observed at land sites did not differ from that transported to the ocean atmosphere (explained in the main text). The f_T is assumed as the mean ratio of the deposition flux of ocean NO_y transported from the land atmosphere (11.0 Tg N yr^{-1}; 5) to the total ocean NO_y deposition (averaging 21.3±1.8 Tg N yr^{-1}, 18.0–23.0 Tg N yr^{-1}; compiled from 5-10). Calculated $\delta^{15}N_{p\text{-}NO3\text{-}E}$ values (Fig. 2) were further used to calculate fractional contributions of NO_x emissions from oil combustion and microbial N cycle in the ocean.

Differences between $\delta^{15}N_{p\text{-}NO3\text{-}}$ and $\delta^{15}N_{i\text{-}NOx}$ values

NO is the initial form of NO_x emissions, but NO will be rapidly oxidized to NO_2 in the atmosphere, forming the photochemical NO_x cycle. The mixing of different NO_x emissions creates the initial NO_x pool in the atmosphere ($i\text{-}NO_x$) (Fig. S1). It is practically challenging, if not impossible, to directly measure the $i\text{-}NO_x$ pool due to its instantaneous emissions and oxidations. However, the transforming fractions of the $i\text{-}NO_x$ to organic and inorganic nitrate components would not differ among NO_x sources. In other words, the fractional contributions of NO_x sources could be preserved in a given form of its products. Besides, observations have shown that organic nitrates account for a low fraction of 9±4% in the land NO_y deposition (data compiled from 11-17). Among inorganic NO_y species in the atmosphere, HONO has significantly lower concentrations than NO_x and $p\text{-}NO_3^-$ (by more than 20 times; Theys et al. (18) because it has a high instability and activity and thus would be

quickly converted to HNO_3 (19) or photolyzed to NO and eventually to p-NO_3^- (20). Thus, we reconstructed the i-NO_x of major inorganic NO_y species (including ambient NO_x, HNO_3, and p-NO_3^-) to differentiate the source contributions, because the conversion of i-NO_x to other compounds and the loss of i-NO_x to other reservoirs/pools would not substantially affect the source contributions and the certainty of our calculations.

The transformation of i-NO_x to HNO_3 and p-NO_3^- (Fig. S1) has distinct N isotopic fractionations (21, 22), which leads to $\delta^{15}N$ differences between ambient NO_x, HNO_3, and p-NO_3^- (23). Accordingly, $\delta^{15}N_{p\text{-}NO3\text{-}}$ values cannot be directly used to differentiate the relative contributions of different NO_x sources. It is crucial to constrain the differences between $\delta^{15}N_{p\text{-}NO3\text{-}}$ and $\delta^{15}N_{i\text{-}NOx}$ values (denoted as the $^{15}\Delta_{i\text{-}NOx\rightarrow p\text{-}NO3\text{-}}$) because this parameter is needed for the source apportionment based on $\delta^{15}N_{p\text{-}NO3\text{-}}$ values on the land and $\delta^{15}N_{p\text{-}NO3\text{-}E}$ values in the ocean (24).

Based on the isotope mass-balance theory, the $\delta^{15}N_{i\text{-}NOx}$ value can be estimated by Eq. 3.

$$\delta^{15}N_{i\text{-}NOx} = (\delta^{15}N_{NOx} \times C_{NO2}/f_{NO2} + \delta^{15}N_{HNO3} \times C_{HNO3} + \delta^{15}N_{p\text{-}NO3\text{-}} \times C_{p\text{-}NO3\text{-}}) / (C_{NO2}/f_{NO2} + C_{HNO3} + C_{p\text{-}NO3\text{-}}) \quad \text{(Eq. 3)}$$

The C_{NO2}, C_{HNO3}, and $C_{p\text{-}NO3\text{-}}$ are concentrations of ambient NO_2, HNO_3, and p-NO_3^-, respectively. f_{NO2} is the fraction of NO_2 in NO_x. $\delta^{15}N_{NOx}$, $\delta^{15}N_{HNO3}$, and $\delta^{15}N_{p\text{-}NO3\text{-}}$ are $\delta^{15}N$ values of NO_x, HNO_3, and p-NO_3^-, respectively. So far, it remains challenging to reconstruct corresponding $\delta^{15}N_{i\text{-}NOx}$ values of p-NO_3^- observed at all land and ocean sites due to the limited data availability of site-specific parameters in Eq. 3. Here we estimated the $\delta^{15}N_{i\text{-}NOx}$ values in two independent scenarios. In Scenario 1, simultaneously observed values of ambient C_{NO2}, C_{HNO3}, $C_{p\text{-}NO3\text{-}}$, $\delta^{15}N_{HNO3}$, and $\delta^{15}N_{p\text{-}NO3\text{-}}$ were used in the calculation of Eq. 3. In Scenario 2, non-synchronously observed values of ambient f_{NO2}, C_{NO2}, C_{HNO3}, $C_{p\text{-}NO3\text{-}}$, $\delta^{15}N_{NOx}$, $\delta^{15}N_{HNO3}$, and $\delta^{15}N_{p\text{-}NO3\text{-}}$ were used in the calculation of Eq. 3. The values and data sources of parameters used for estimating $\delta^{15}N_{i\text{-}NOx}$ values are shown in Table S3. Then, we calculated the corresponding $^{15}\Delta_{i\text{-}NOx\rightarrow p\text{-}NO3\text{-}}$ values in Scenarios 1 and 2 (Eq. 4), and their mean

values were used to estimate source contributions in Eqs. 5 & 6 due to tiny differences between the two scenarios (Fig. S2).

$$^{15}\Delta_{\text{i-NOx}\rightarrow\text{p-NO3-}} = \delta^{15}N_{\text{p-NO3-}} - \delta^{15}N_{\text{i-NOx}} \quad \text{(Eq. 4)}$$

Source contribution analyses

The $\delta^{15}N_{\text{p-NO3-}}$ is determined by fractional contributions and $\delta^{15}N$ values of corresponding NO_x sources and the $^{15}\Delta_{\text{i-NOx}\rightarrow\text{p-NO3-}}$. Here we estimated the relative contributions of coal combustion (S1), oil combustion (S2), biomass burning (S3), and microbial N cycle (S4) to the p-NO_3^- in the land (Eq. 5) and S2 and S4 to p-NO_3^- in the ocean (Eq. 6) by using isotope mass-balance methods.

$$\delta^{15}N_{\text{p-NO3-L}} = f_{S1} \times \delta^{15}N_{S1} + f_{S2} \times \delta^{15}N_{S2} + f_{S3} \times \delta^{15}N_{S3} + f_{S4} \times \delta^{15}N_{S4}) + {}^{15}\Delta_{\text{i-NOx}\rightarrow\text{p-NO3-}} \quad \text{(Eq. 5)}$$

$$\delta^{15}N_{\text{p-NO3-E}} = (F_{S2} \times \delta^{15}N_{S2} + F_{S4} \times \delta^{15}N_{S4}) + {}^{15}\Delta_{\text{i-NOx}\rightarrow\text{p-NO3-}} \quad \text{(Eq. 6)}$$

In Eq. 5, f_{S1}, f_{S2}, f_{S3}, and f_{S4} are relative contributions of NO_x from S1, S2, S3, and S4 to p-NO_3^- in the land, respectively, and we assumed that $f_{S1} + f_{S2} + f_{S3} + f_{S4} = 1$. In Eq. 6, F_{S2} and F_{S4} are relative contributions of NO_x from S2 and S4 to p-NO_3^- in the ocean, respectively, and we assumed that $F_{S2} + F_{S4} = 1$. The $\delta^{15}N_{S1}$, $\delta^{15}N_{S2}$, $\delta^{15}N_{S3}$, and $\delta^{15}N_{S4}$ represent $\delta^{15}N$ values of NO_x from S1, S2, S3, and S4, respectively (Table S2).

For the $\delta^{15}N_{S2}$, we synthesized the existing data and assigned the same mean±SD value for all land and ocean sites based on two major reasons. Firstly, the $\delta^{15}N$ values of tailpipe NO_x from individual vehicles measured by active sampling techniques did not differ between observations in China (-8.5±4.3‰, -18.8‰ – 6.4‰) (25, 26) and USA (-7.7±5.9‰, -20.9‰ – 9.8‰) (27-29). Secondly, $\delta^{15}N$ values of on-road NO_x from vehicles in the USA (-4.6±1.7‰, -0.9‰ – -8.3‰) (30, 31) were all in the range of tailpipe $\delta^{15}N$-NO_x values (-20.9‰ – 9.8‰). The same $\delta^{15}N_{S3}$ values can be assigned for land sites. In our study, sites of land $\delta^{15}N_{\text{p-NO3-}}$ observations are mainly distributed between 15°N - 60°N (Fig. 1), where leaf $\delta^{15}N$ values (y) decreased with the latitudes (x) ($y = -0.07728x + 1.7945$, Fig. S6) (32). Based on the linear

relationship between $\delta^{15}N_{S3}$ (y) and the $\delta^{15}N$ of biomass (x) ($y = 0.48825x + 0.12617$, Fig. S7) (33-36), the maximum latitudinal variation of leaf $\delta^{15}N$ values (3.5‰) would cause a $\delta^{15}N_{S3}$ difference of 1.7‰, even if the leaf $\delta^{15}N$ (often more variable than the wood $\delta^{15}N$; 37, 38) can represent the corresponding $\delta^{15}N$ of biomass. So far, the $\delta^{15}N_{S3}$ values did not differ between observations in China (-0.5±2.5‰) and the USA (1.1±2.8‰) (Fig. S8). Accordingly, the mean±SD of $\delta^{15}N_{S3}$ values was used in our calculations due to its low spatial variability. The $\delta^{15}N_{S4}$ values had a wide range (Fig. S9) but showed no apparent variations among emissions from a single source nor differences between measuring techniques. Accordingly, we used their mean±SD value for both land and ocean sites because it was distinctly lower than other NO_x sources.

The f_{S1}–f_{S4}, $F_{S2,}$ and F_{S4} values were calculated using a Bayesian isotope-mixing model (named Stable Isotope Analysis in R, SIAR). The SIAR model (39) uses a Bayesian framework to establish a logical prior distribution based on Dirichlet distribution (40) for estimating source contributions (f_{S1}–f_{S4} for the land, F_{S2} and F_{S4} for the ocean). It has the potential to provide reliable estimations of source contributions because the isotope effect (i.e., $^{15}\Delta_{i\text{-}NOx\rightarrow p\text{-}NO3\text{-}}$ values in this study) and variability in $\delta^{15}N$ values of both sources (i.e., $\delta^{15}N$ values of NO_x from S1−S4 in this study) and mixture (i.e., $\delta^{15}N_{p\text{-}NO3\text{-}L}$ and $\delta^{15}N_{p\text{-}NO3\text{-}E}$ values in this study) (41, 42) have been considered in this model. The SIAR model has been widely used to quantify the relative contributions of multiple NO_x emission sources to p-NO_3^- (43-46). In each run of the SIAR model, the mean±SD of source $\delta^{15}N$ values (Table S2), the mean±SD of $^{15}\Delta_{i\text{-}NOx\rightarrow p\text{-}NO3\text{-}}$ values (Fig. S2), and site-based mean $\delta^{15}N_{p\text{-}NO3\text{-}L}$ or $\delta^{15}N_{p\text{-}NO3\text{-}E}$ values (Fig. 2) were input into the model. In each run, the percentage data of each source (n=10000) output from the SIAR model were used to calculate the mean±SD value of the corresponding source (Fig. S3a).

Emission amounts of dominant NO_x sources

Based on the emission amount of NO_x from fossil fuel sources (i.e., the sum of S1 and S2) in the land (28.4±1.8 Tg N yr^{-1} Tg N yr^{-1}) (47-51) and its relative contribution (i.e.,

the sum of f_{S1} and f_{S2} values), the total amount of land NO_x emissions ($E_{total\text{-}land}$) was calculated by using Eq. 7.

$$E_{total\text{-}land} = (28.4 \pm 1.8) / (f_{S1} + f_{S2}) \qquad \text{(Eq. 7)}$$

Based on the $E_{total\text{-}land}$, f_{S1}, f_{S2}, f_{S3}, and f_{S4} values, the amount of land NO_x emissions from S1 ($E_{S1\text{-}land}$), S2 ($E_{S2\text{-}land}$), S3 ($E_{S3\text{-}land}$), and S4 ($E_{S4\text{-}land}$) was calculated by using Eqs. 8–11, respectively.

$$E_{S1\text{-}land} = E_{total\text{-}land} \times f_{S1} \qquad \text{(Eq. 8)}$$

$$E_{S2\text{-}land} = E_{total\text{-}land} \times f_{S2} \qquad \text{(Eq. 9)}$$

$$E_{S3\text{-}land} = E_{total\text{-}land} \times f_{S3} \qquad \text{(Eq. 10)}$$

$$E_{S4\text{-}land} = E_{total\text{-}land} \times f_{S4} \qquad \text{(Eq. 11)}$$

Based on the F_{S2} value and the NO_x emission from oil combustion in the ocean ($E_{S2\text{-}ocean}$) (6.4±0.8 Tg N yr^{-1} Tg N yr^{-1}; data compiled from 52-58), the total ocean NO_x emission ($E_{total\text{-}ocean}$) was calculated by Eq. 12.

$$E_{total\text{-}ocean} = (6.4 \pm 0.8) / F_{S2} \qquad \text{(Eq. 12)}$$

Based on the $E_{total\text{-}ocean}$ and F_{S4} values, the ocean microbial NO_x emission ($E_{S4\text{-}ocean}$) was calculated by Eq. 13.

$$E_{S4\text{-}ocean} = E_{total\text{-}ocean} \times F_{S4} \qquad \text{(Eq. 13)}$$

Further, we calculated the amounts of global NO_x emissions from S1 ($E_{S1\text{-}global}$), S2 ($E_{S2\text{-}global}$), S3 ($E_{S3\text{-}global}$), S4 ($E_{S4\text{-}global}$), and all four sources ($E_{total\text{-}global}$) as follows.

$$E_{S1\text{-}global} = E_{S1\text{-}land} \qquad \text{(Eq. 14)}$$

$$E_{S2\text{-}global} = E_{S2\text{-}land} + E_{S2\text{-}ocean} \qquad \text{(Eq. 15)}$$

$$E_{S3\text{-}global} = E_{S3\text{-}land} \qquad \text{(Eq. 16)}$$

$$E_{S4\text{-}global} = E_{S4\text{-}land} + E_{S4\text{-}ocean} \quad \text{(Eq. 17)}$$

$$E_{total\text{-}global} = E_{total\text{-}land} + E_{total\text{-}ocean} \quad \text{(Eq. 18)}$$

The SD values of parameters in Eqs. 1−18 were propagated by using the Monte Carlo method to estimate the uncertainties (SD values) of the calculated results in each calculation. The one-way analyses of variance were performed to examine the $\delta^{15}N$ difference, and the significance level was set as $p<0.05$ (Fig. 2).

Supplementary Figures

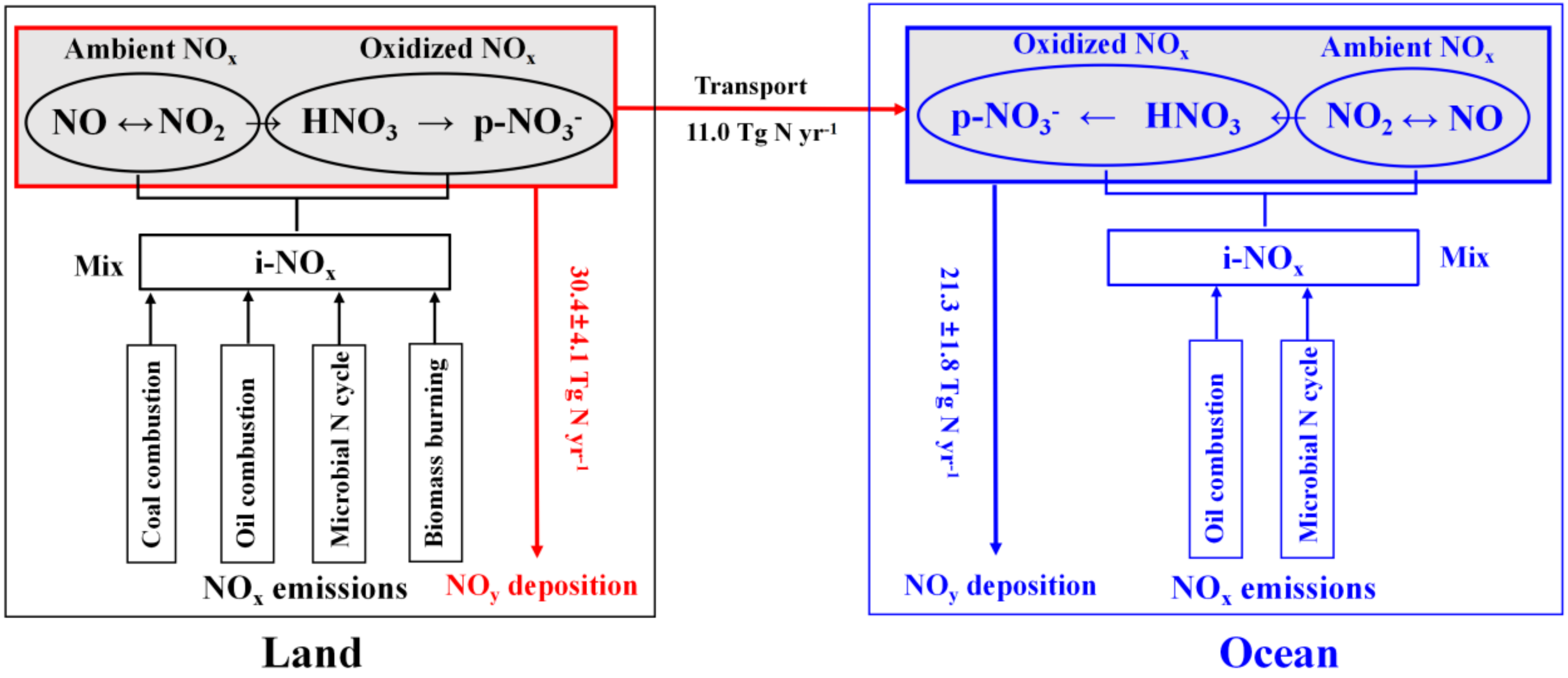

Fig. S1. A schematic map showing NO_x emissions, transformations, and deposition in the land and ocean atmosphere. The i-NO_x represents the mixed pool of different NO_x emission sources. The amounts of NO_y transported from the land to the ocean atmosphere and NO_y deposition in the ocean were cited from the literature of 5-10 (Detailed in Methods). The NO_y deposition in the land was estimated by using the global NO_y deposition minus the NO_y deposition in the ocean. The global NO_y deposition was cited from the literature of 5, 8, 59-62.

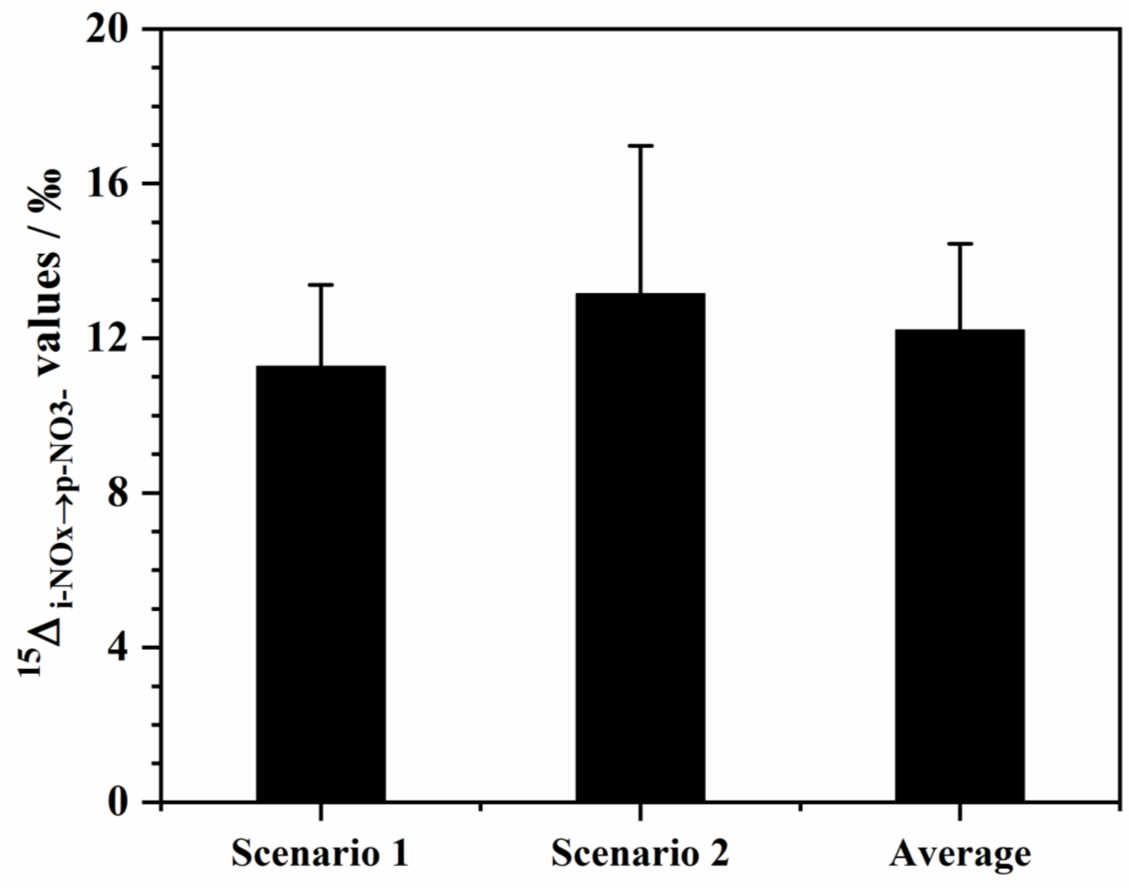

Fig. S2. Differences in $\delta^{15}N$ values between p-NO_3^- and i-NO_x in the atmosphere ($^{15}\Delta_{i\text{-}NOx \rightarrow p\text{-}NO3\text{-}}$ values). Mean ± SD values are shown. Scenarios 1 & 2 show estimations based on simultaneous and non-synchronous observations of atmospheric NO_x, HNO_3, and p-NO_3^- (detailed in Methods). Average: the mean value of mean values of Scenarios 1 & 2.

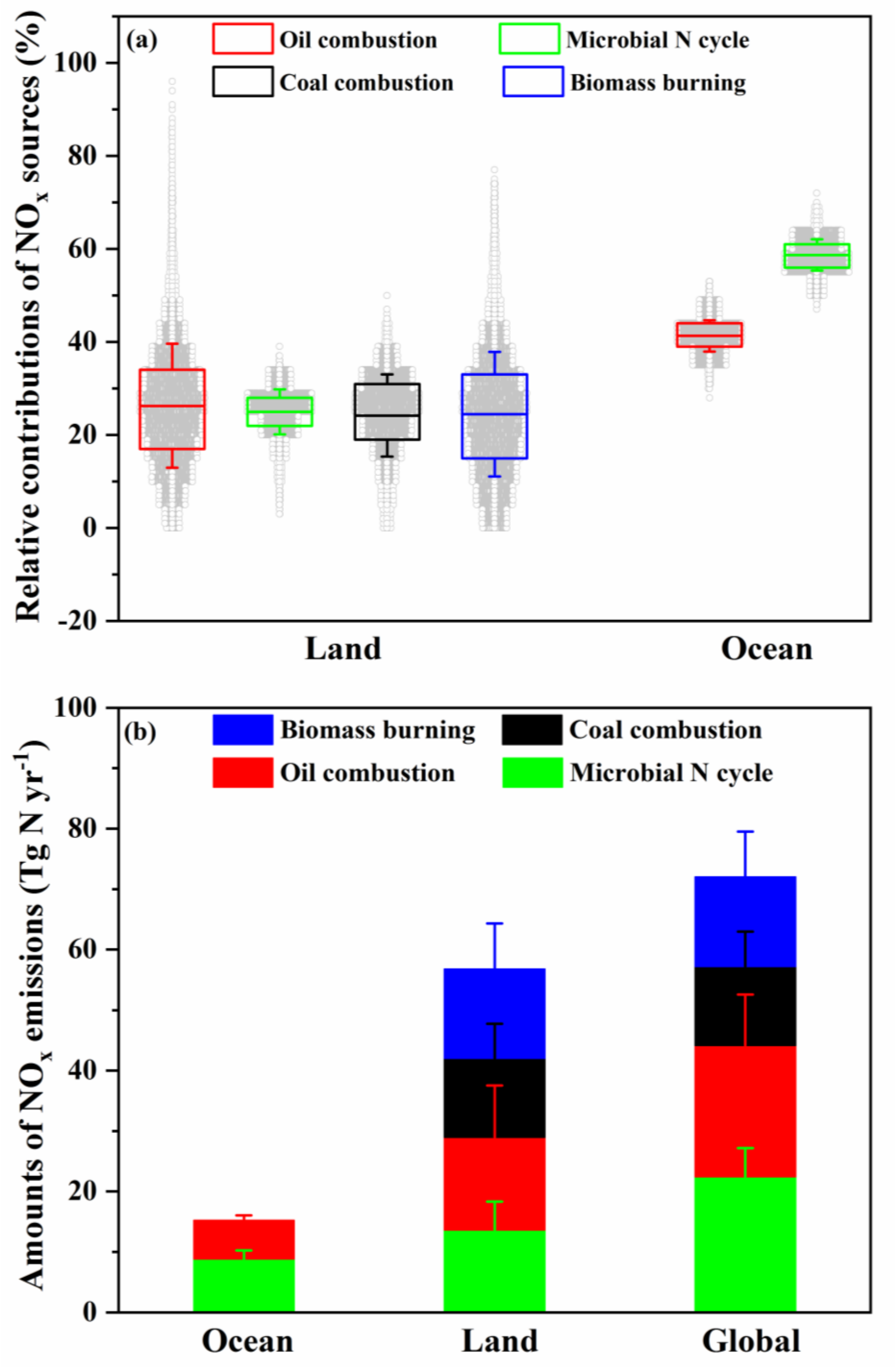

Fig. S3. Fractional contributions (a) and amounts (b) of dominant sources to land and ocean NO_x emissions. In panel a, circles around each box (n = 10^4) show the percentage values output from the SIAR model. The box encompasses the 25th–75th percentiles, whiskers and lines in boxes are standard deviations and arithmetic mean values, respectively. In panel b, values are mean ± SD (calculations were detailed in Methods).

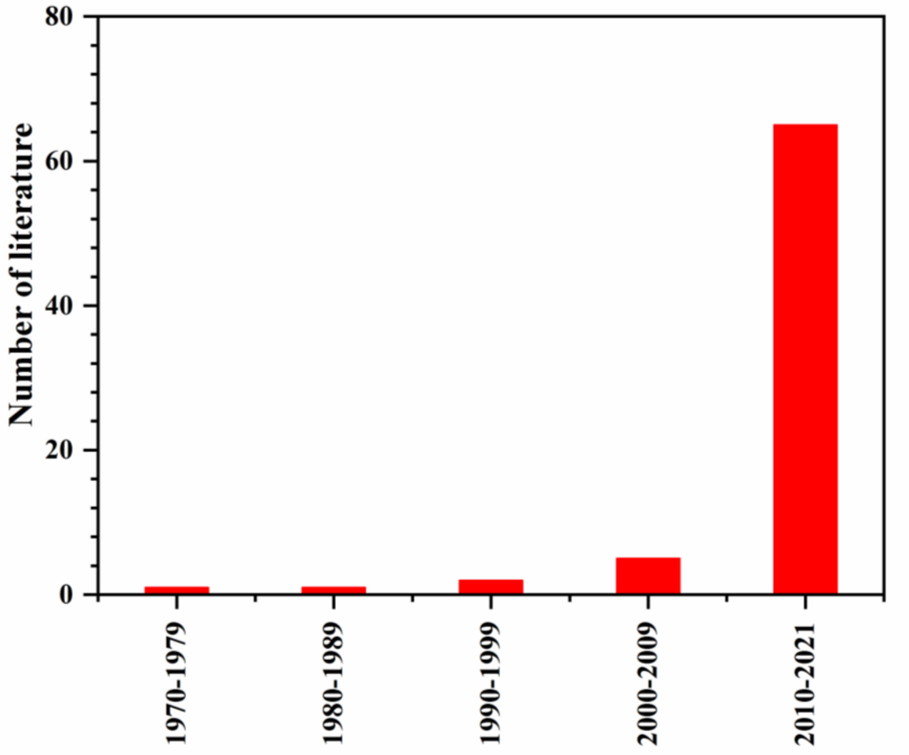

Fig. S4. Distributions of publications with $\delta^{15}N_{p\text{-}NO3\text{-}}$ observations from 1970 to 2021.

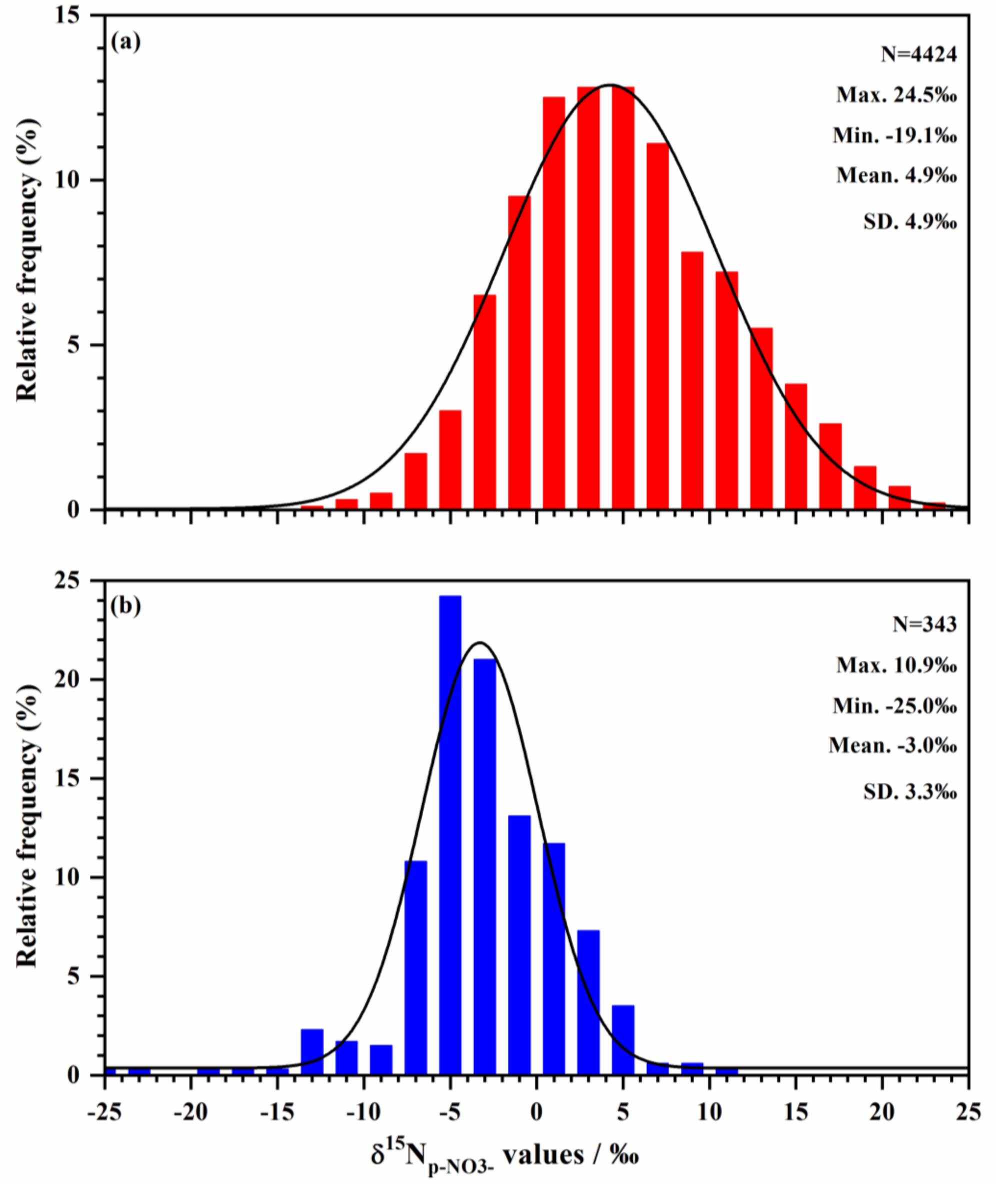

Fig. S5. Relative frequency histograms of $\delta^{15}N_{p\text{-}NO3\text{-}}$ values at land (a) and ocean (b) sites.

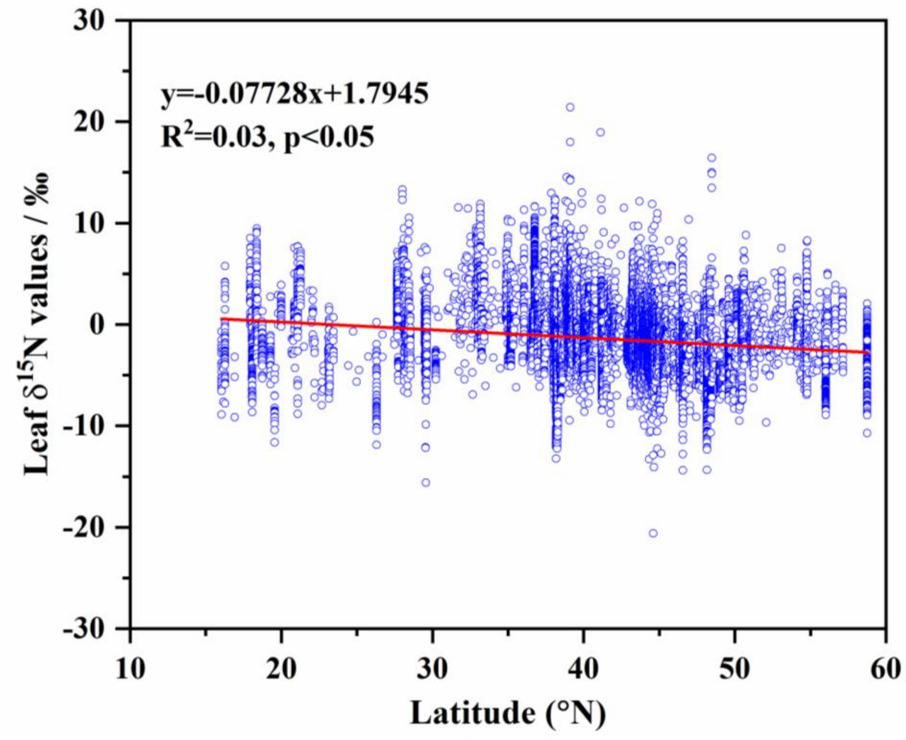

Fig. S6. The variation of leaf $\delta^{15}N$ values with the latitudes in the northern hemisphere. Data was cited from Craine et al (32).

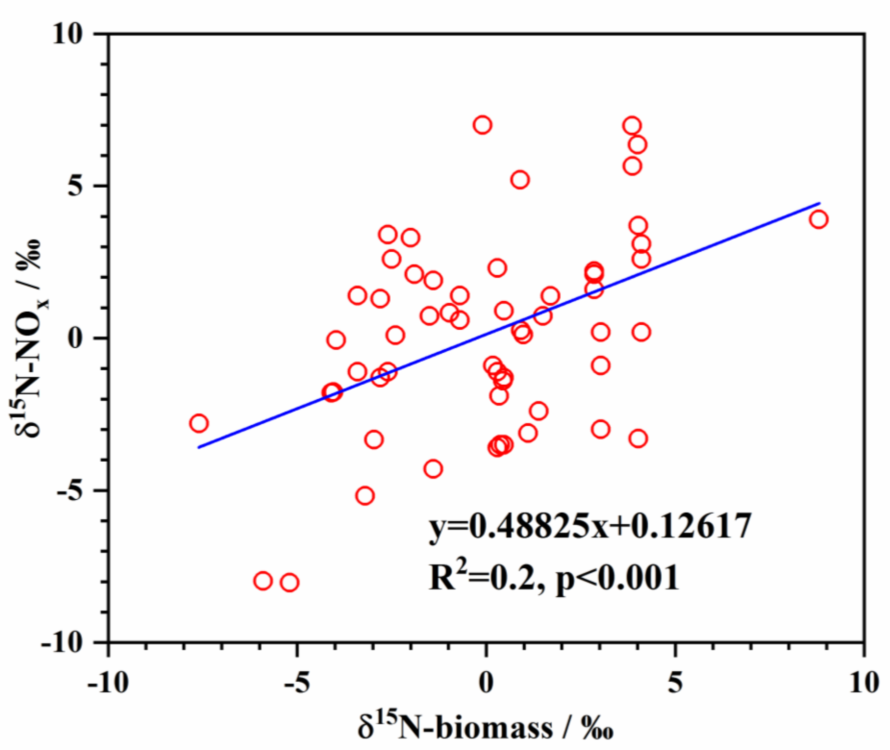

Fig. S7. The $\delta^{15}N$ relationship between NO_x produced by biomass burning and N in corresponding biomass. Data was cited from refs 33-36.

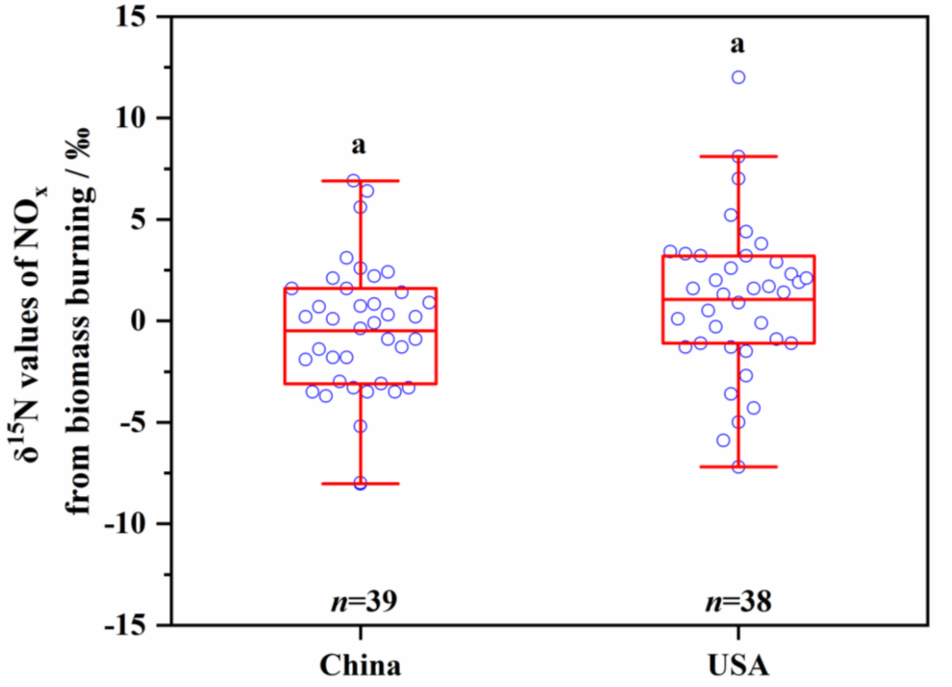

Fig. S8. $\delta^{15}N$ values of NO_x from biomass burning observed in China and USA. The box encompasses the 25th–75th percentiles, whiskers and lines in boxes are the SD and mean values, respectively. Different letters above the boxes show significant differences at $p<0.05$.

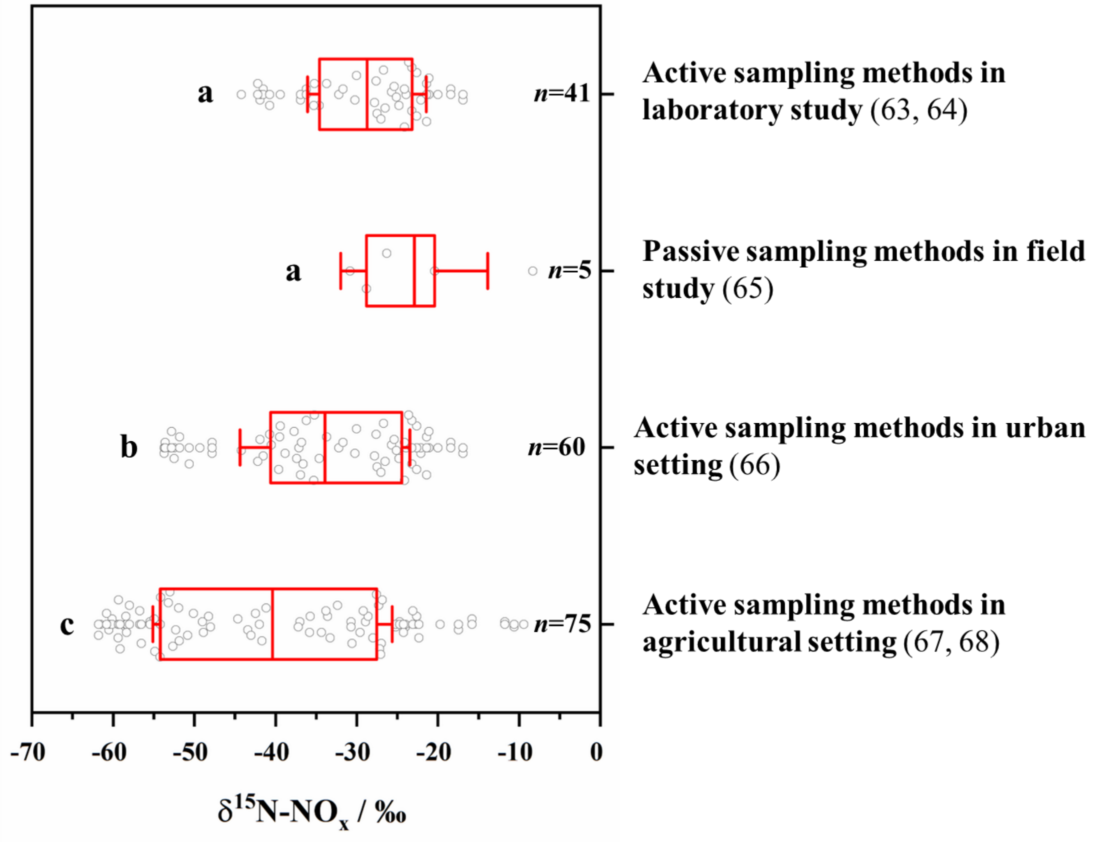

Fig. S9. δ^{15}N values of NO_x from microbial N cycles in different settings based on different sampling methods. Each box encompasses the 25th–75th percentiles, whiskers and lines in boxes are the SD and mean values, respectively. Different letters above the boxes show significant differences at $p<0.05$.

Supplementary Tables

Table S1. Site and sample information, data, and references of $\delta^{15}N_{p\text{-}NO3\text{-}}$ observations on the land and ocean. Site distributions are shown in Fig. 1, and full references are listed in Text S2.

Latitude (°)	Longitude (°)	Site types	Sample types	Replicates	$\delta^{15}N_{p\text{-}NO3\text{-}}$ values / ‰				References
					Mean	SD	Min	Max	
-57.82	104.80	Ocean	Aerosol	1	-25.0				Shi et al., 2021 Earth Planet. Sc. Lett.
-54.20	112.41	Ocean	Aerosol	1	-12.9				Shi et al., 2021 Earth Planet. Sc. Lett.
-53.05	73.74	Ocean	Aerosol	1	-19.1				Shi et al., 2021 Earth Planet. Sc. Lett.
-49.22	113.76	Ocean	Aerosol	1	-12.4				Shi et al., 2021 Earth Planet. Sc. Lett.
-49.08	79.80	Ocean	Aerosol	1	-23.9				Shi et al., 2021 Earth Planet. Sc. Lett.
-43.64	111.87	Ocean	Aerosol	1	-14.7				Shi et al., 2021 Earth Planet. Sc. Lett.
-41.41	91.48	Ocean	Aerosol	1	-10.9				Shi et al., 2021 Earth Planet. Sc. Lett.
-41.33	174.75	Land	Aerosol	34	-4.5	3.6	-12.6	6.2	Li et al., 2021 Atmos. Environ.
-38.49	113.41	Ocean	Aerosol	1	-5.1				Shi et al., 2021 Earth Planet. Sc. Lett.
-37.00	-172.00	Ocean	Aerosol	1	-5.0				Kamezaki et al., 2019 Atmos. Environ.
-35.69	103.52	Ocean	Aerosol	1	-5.9				Shi et al., 2021 Earth Planet. Sc. Lett.
-34.06	115.09	Ocean	Aerosol	1	-4.2				Shi et al., 2021 Earth Planet. Sc. Lett.
-32.85	112.80	Ocean	Aerosol	1	-9.8				Shi et al., 2021 Earth Planet. Sc. Lett.
-32.04	115.71	Ocean	Aerosol	1	-2.1				Shi et al., 2021 Earth Planet. Sc. Lett.
-31.04	114.98	Ocean	Aerosol	1	-5.6				Shi et al., 2021 Earth Planet. Sc. Lett.
-30.00	-169.00	Ocean	Aerosol	1	-5.8				Kamezaki et al., 2019 Atmos. Environ.

-27.19	113.40	Ocean	Aerosol	1	-5.4				Shi et al., 2021 Earth Planet. Sc. Lett.
-27.10	12.00	Ocean	Aerosol	1	-6.0				Morin et al., 2009 J. Geophys. Res.
-25.75	28.25	Land	Aerosol	12	-6.2	3.5	-2.1	12.1	Heaton 1987 Atmos. Environ.
-23.34	-69.97	Land	Aerosol	1	7.0				Wang et al., 2014 Geochim. Cosmochim. Ac.
-23.14	-70.51	Land	Aerosol	1	8.4				Wang et al., 2014 Geochim. Cosmochim. Ac.
-23.10	7.47	Ocean	Aerosol	1	-4.2				Morin et al., 2009 J. Geophys. Res.
-23.10	-67.43	Land	Aerosol	1	10.5				Wang et al., 2014 Geochim. Cosmochim. Ac.
-23.07	-69.39	Land	Aerosol	1	3.6				Wang et al., 2014 Geochim. Cosmochim. Ac.
-23.00	-69.81	Land	Aerosol	1	9.4				Wang et al., 2014 Geochim. Cosmochim. Ac.
-22.97	-68.35	Land	Aerosol	1	1.2				Wang et al., 2014 Geochim. Cosmochim. Ac.
-22.85	-69.69	Land	Aerosol	1	6.1				Wang et al., 2014 Geochim. Cosmochim. Ac.
-22.81	-69.07	Land	Aerosol	1	2.1				Wang et al., 2014 Geochim. Cosmochim. Ac.
-22.70	-68.49	Land	Aerosol	1	2.6				Wang et al., 2014 Geochim. Cosmochim. Ac.
-21.96	113.91	Ocean	Aerosol	1	-16.1				Shi et al., 2021 Earth Planet. Sc. Lett.
-21.40	112.94	Ocean	Aerosol	1	-6.7				Shi et al., 2021 Earth Planet. Sc. Lett.
-21.00	-169.00	Ocean	Aerosol	1	-1.3				Kamezaki et al., 2019 Atmos. Environ.
-19.30	3.78	Ocean	Aerosol	1	-6.7				Morin et al., 2009 J. Geophys. Res.
-16.00	-104.00	Ocean	Aerosol	1	-3.5				Carter et al., 2021 ACS Earth Space Chem.
-15.20	0.65	Ocean	Aerosol	1	-1.3				Morin et al., 2009 J. Geophys. Res.
-14.99	-109.19	Ocean	Aerosol	1	-3.2				Carter et al., 2021 ACS Earth Space Chem.
-14.98	-112.75	Ocean	Aerosol	1	-5.5				Carter et al., 2021 ACS Earth Space Chem.

-14.77	-114.99	Ocean	Aerosol	1	-4.4	Carter et al., 2021 ACS Earth Space Chem.
-14.00	-120.00	Ocean	Aerosol	2	-3.3	Carter et al., 2021 ACS Earth Space Chem.
-14.00	-170.00	Ocean	Aerosol	1	-3.3	Kamezaki et al., 2019 Atmos. Environ.
-14.00	-99.00	Ocean	Aerosol	2	-5.6	Carter et al., 2021 ACS Earth Space Chem.
-12.93	114.89	Ocean	Aerosol	1	-4.5	Shi et al., 2021 Earth Planet. Sc. Lett.
-12.54	-124.99	Ocean	Aerosol	1	-8.0	Carter et al., 2021 ACS Earth Space Chem.
-12.05	-77.66	Ocean	Aerosol	2	-4.7	Carter et al., 2021 ACS Earth Space Chem.
-12.01	-79.20	Ocean	Aerosol	2	-5.8	Carter et al., 2021 ACS Earth Space Chem.
-12.00	-94.00	Ocean	Aerosol	1	-7.1	Carter et al., 2021 ACS Earth Space Chem.
-12.00	-81.50	Ocean	Aerosol	2	-3.8	Carter et al., 2021 ACS Earth Space Chem.
-12.00	-86.50	Ocean	Aerosol	2	-5.4	Carter et al., 2021 ACS Earth Space Chem.
-11.67	-128.00	Ocean	Aerosol	2	-12.5	Carter et al., 2021 ACS Earth Space Chem.
-11.60	-135.00	Ocean	Aerosol	1	-11.8	Carter et al., 2021 ACS Earth Space Chem.
-11.40	-2.20	Ocean	Aerosol	1	-4.5	Morin et al., 2009 J. Geophys. Res.
-11.31	115.25	Ocean	Aerosol	1	-9.4	Shi et al., 2021 Earth Planet. Sc. Lett.
-11.31	-140.00	Ocean	Aerosol	2	-13.0	Carter et al., 2021 ACS Earth Space Chem.
-11.03	-142.95	Ocean	Aerosol	2	-11.7	Carter et al., 2021 ACS Earth Space Chem.
-11.00	164.00	Ocean	Aerosol	1	-1.5	Kamezaki et al., 2019 Atmos. Environ.
-10.50	-152.00	Ocean	Aerosol	1	-9.4	Carter et al., 2021 ACS Earth Space Chem.
-7.88	-6.17	Ocean	Aerosol	1	-1.3	Morin et al., 2009 J. Geophys. Res.
-4.59	117.59	Ocean	Aerosol	1	-6.4	Shi et al., 2021 Earth Planet. Sc. Lett.

-4.55	-10.10	Ocean	Aerosol	1	-2.3	Morin et al., 2009 J. Geophys. Res.
-2.41	118.83	Ocean	Aerosol	1	-3.6	Shi et al., 2021 Earth Planet. Sc. Lett.
-2.18	-13.50	Ocean	Aerosol	1	-3.4	Morin et al., 2009 J. Geophys. Res.
0.22	-23.22	Ocean	Aerosol	1	-4.6	Baker et al., 2007 Deep-Sea Res. Pt. I
0.22	-25.84	Ocean	Aerosol	1	2.0	Baker et al., 2007 Deep-Sea Res. Pt. I
0.83	120.83	Ocean	Aerosol	1	-0.1	Shi et al., 2021 Earth Planet. Sc. Lett.
1.89	-23.49	Ocean	Aerosol	1	-6.2	Baker et al., 2007 Deep-Sea Res. Pt. I
3.28	-26.20	Ocean	Aerosol	1	2.8	Baker et al., 2007 Deep-Sea Res. Pt. I
3.53	-5.76	Ocean	Aerosol	1	-3.0	Baker et al., 2007 Deep-Sea Res. Pt. I
3.72	-10.26	Ocean	Aerosol	1	-3.6	Baker et al., 2007 Deep-Sea Res. Pt. I
5.00	-178.00	Ocean	Aerosol	1	-11.4	Kamezaki et al., 2019 Atmos. Environ.
5.19	-19.50	Ocean	Aerosol	1	-2.7	Morin et al., 2009 J. Geophys. Res.
5.30	-13.53	Ocean	Aerosol	1	-7.3	Baker et al., 2007 Deep-Sea Res. Pt. I
5.81	125.00	Ocean	Aerosol	1	-2.3	Shi et al., 2021 Earth Planet. Sc. Lett.
5.96	-24.12	Ocean	Aerosol	1	2.1	Baker et al., 2007 Deep-Sea Res. Pt. I
6.00	-170.00	Ocean	Aerosol	1	-2.4	Kamezaki et al., 2019 Atmos. Environ.
6.76	124.17	Ocean	Aerosol	1	-3.2	Shi et al., 2021 Earth Planet. Sc. Lett.
6.89	-26.27	Ocean	Aerosol	1	1.3	Baker et al., 2007 Deep-Sea Res. Pt. I
7.00	176.00	Ocean	Aerosol	1	-13.0	Kamezaki et al., 2019 Atmos. Environ.
7.16	-17.17	Ocean	Aerosol	1	-10.9	Baker et al., 2007 Deep-Sea Res. Pt. I
8.00	155.00	Ocean	Aerosol	1	-3.00	Kamezaki et al., 2019 Atmos. Environ.

8.28	-24.57	Ocean	Aerosol	1	-6.1	Baker et al., 2007 Deep-Sea Res. Pt. I
8.76	-20.40	Ocean	Aerosol	1	-4.1	Morin et al., 2009 J. Geophys. Res.
9.01	-18.94	Ocean	Aerosol	1	-2.8	Baker et al., 2007 Deep-Sea Res. Pt. I
9.71	-51.38	Ocean	Aerosol	1	-6.5	Baker et al., 2007 Deep-Sea Res. Pt. I
9.88	-41.72	Ocean	Aerosol	1	0.9	Baker et al., 2007 Deep-Sea Res. Pt. I
9.89	-48.00	Ocean	Aerosol	1	-2.9	Baker et al., 2007 Deep-Sea Res. Pt. I
9.95	-30.28	Ocean	Aerosol	1	-0.9	Baker et al., 2007 Deep-Sea Res. Pt. I
9.96	-36.47	Ocean	Aerosol	1	-1.1	Baker et al., 2007 Deep-Sea Res. Pt. I
9.97	-38.72	Ocean	Aerosol	1	-1.9	Baker et al., 2007 Deep-Sea Res. Pt. I
9.98	-44.81	Ocean	Aerosol	1	-3.4	Baker et al., 2007 Deep-Sea Res. Pt. I
10.00	174.00	Ocean	Aerosol	1	0.2	Kamezaki et al., 2019 Atmos. Environ.
10.04	-27.47	Ocean	Aerosol	1	1.0	Baker et al., 2007 Deep-Sea Res. Pt. I
10.24	-33.19	Ocean	Aerosol	1	-3.3	Baker et al., 2007 Deep-Sea Res. Pt. I
10.45	-53.72	Ocean	Aerosol	1	0.41	Baker et al., 2007 Deep-Sea Res. Pt. I
10.59	-20.71	Ocean	Aerosol	1	-4.1	Baker et al., 2007 Deep-Sea Res. Pt. I
10.77	-19.03	Ocean	Aerosol	1	-0.93	Baker et al., 2007 Deep-Sea Res. Pt. I
10.77	-21.65	Ocean	Aerosol	1	-6.3	Baker et al., 2007 Deep-Sea Res. Pt. I
10.78	-24.74	Ocean	Aerosol	1	-5.5	Baker et al., 2007 Deep-Sea Res. Pt. I
11.18	126.95	Ocean	Aerosol	1	-3.5	Shi et al., 2021 Earth Planet. Sc. Lett.
11.23	-18.55	Ocean	Aerosol	1	-3.64	Baker et al., 2007 Deep-Sea Res. Pt. I
12.80	-21.00	Ocean	Aerosol	1	-2.7	Morin et al., 2009 J. Geophys. Res.

16.66	126.62	Ocean	Aerosol	1	0.3				Shi et al., 2021 Earth Planet. Sc. Lett.
16.83	112.33	Ocean	TSP	62	1.3	1.3	-2.6	4.8	Xiao et al., 2015 Atmos. Environ.
16.85	-24.87	Ocean	Aerosol	58	-5.5	0.9	-8.	-2.8	Savarino et al., 2013 PNAS
17.10	-20.20	Ocean	Aerosol	1	-5.5				Morin et al., 2009 J. Geophys. Res.
17.14	126.60	Ocean	Aerosol	1	-4.8				Shi et al., 2021 Earth Planet. Sc. Lett.
18.00	162.00	Ocean	Aerosol	1	-0.3				Kamezaki et al., 2019 Atmos. Environ.
18.09	116.53	Ocean	TSP	1	-1.2				Xiao et al., 2015 Atmos. Environ.
18.28	114.03	Ocean	TSP	1	6.5				Xiao et al., 2015 Atmos. Environ.
19.41	119.69	Ocean	TSP	1	0.8				Xiao et al., 2015 Atmos. Environ.
19.80	-16.80	Ocean	Aerosol	1	-5.5				Morin et al., 2009 J. Geophys. Res.
20.00	-169.00	Ocean	Aerosol	1	-2.3				Kamezaki et al., 2019 Atmos. Environ.
20.70	116.72	Ocean	Aerosol	72	-2.9	1.8	-7.5	4.1	Yang et al., 2014 Atmos. Chem. Phys.
21.00	-157.00	Ocean	Aerosol	1	-2.1				Kamezaki et al., 2019 Atmos. Environ.
21.59	114.91	Ocean	TSP	1	3.8				Xiao et al., 2015 Atmos. Environ.
21.75	115.25	Ocean	TSP	1	5.8				Xiao et al., 2015 Atmos. Environ.
21.93	113.71	Ocean	TSP	1	6.2				Xiao et al., 2015 Atmos. Environ.
22.00	119.90	Ocean	TSP	1	5.7				Xiao et al., 2015 Atmos. Environ.
22.00	157.00	Ocean	Aerosol	1	2.3				Kamezaki et al., 2019 Atmos. Environ.
22.10	114.00	Ocean	TSP	1	5.6				Xiao et al., 2015 Atmos. Environ.
22.16	113.54	Ocean	TSP	1	3.7				Xiao et al., 2015 Atmos. Environ.
22.13	113.82	Ocean	TSP	1	5.6				Xiao et al., 2015 Atmos. Environ.

22.20	-15.70	Ocean	Aerosol	1	-3.3				Morin et al., 2009 J. Geophys. Res.
22.65	115.55	Ocean	TSP	1	5.7				Xiao et al., 2015 Atmos. Environ.
22.70	112.91	Land	$PM_{2.5}$	68	8.0	2.2	2.6	15.4	Su et al., 2020 Atmos. Environ.
22.71	113.68	Ocean	TSP	1	8.2				Xiao et al., 2015 Atmos. Environ.
22.75	116.75	Ocean	TSP	1	5.0				Xiao et al., 2015 Atmos. Environ.
22.77	126.17	Ocean	Aerosol	1	-4.0				Shi et al., 2021 Earth Planet. Sc. Lett.
22.83	108.28	Land	$PM_{2.5}$	167	-2.9	1.9	-5.7	10.1	Guo et al., 2021 Environ. Res.
22.90	113.62	Ocean	TSP	1	-7.7				Xiao et al., 2015 Atmos. Environ.
23.08	113.04	Ocean	TSP	2	7.5	3.4	4.1	10.9	Xiao et al., 2015 Atmos. Environ.
23.15	113.36	Land	$PM_{2.5}$	94	-3.5	4.7	-12.7	18.0	Zong et al., 2020 Environ. Int.
23.47	120.87	Land	TSP	148	-3.1	2.8	-16.6	6.5	Guha et al., 2017 Atmos. Environ.
24.50	-14.30	Ocean	Aerosol	1	-2.3				Morin et al., 2009 J. Geophys. Res.
25.06	102.70	Land	$PM_{2.5}$	174	7.2	2.1	0.6	15.4	Guo et al., 2021 Environ. Res.
26.00	144.00	Ocean	Aerosol	1	-0.6				Kamezaki et al., 2019 Atmos. Environ.
26.57	106.72	Land	$PM_{2.5}$	74	1.7	3.8	-7.7	10.7	Li 2017 Master thesis; Li et al., 2021 Atmos. Res.
26.81	124.52	Ocean	Aerosol	1	-1.9				Shi et al., 2021 Earth Planet. Sc. Lett.
27.90	-14.00	Ocean	Aerosol	1	-2.6				Morin et al., 2009 J. Geophys. Res.
28.36	86.95	Land	TSP	36	-5.0	1.92	-10.6	-0.8	Wang et al., 2020 Environ. Pollut.; Lin et al., 2021 Environ. Sci. Technol.
28.37	123.80	Ocean	Aerosol	1	4.9				Shi et al., 2021 Earth Planet. Sc. Lett.
28.40	87.00	Land	Aerosol	6	-2.5	0.9	-3.9	-0.8	Lin et al., 2020 J. Geophys. Res.-Atmos.

28.70	115.80	Land	$PM_{2.5}$	99	4.9	3.1	-1.9	13.8	Luo et al., 2020 Atmos. Environ.
28.70	115.90	Land	$PM_{2.5}$	196	5.1	3.1	-10.5	13.2	Xiao et al., 2020 J. Geophys. Res.-Atmos.; Zhang et al., 2020 Atmos. Environ.
29.00	147.00	Ocean	Aerosol	1	-5.7				Kamezaki et al., 2019 Atmos. Environ.
29.52	34.92	Land	TSP	51	-1.3	1.8	-5.6	3.5	Wankel et al., 2010 Mar. Chem.
30.00	-176.00	Ocean	Aerosol	1	-1.2				Kamezaki et al., 2019 Atmos. Environ.
30.40	122.50	Land	TSP	58	6.4	3.7	-2.8	24.5	Fang 2020 Master thesis
30.48	120.28	Land	$PM_{2.5}$	12	5.9	2.1	1.1	9.2	Jin et al., 2020 Atmos. Environ.
30.50	-13.50	Ocean	Aerosol	1	-2.3				Morin et al., 2009 J. Geophys. Res.
30.52	104.36	Land	$PM_{2.5}$	91	6.6	5.0	-5.6	20.0	Zong et al., 2020 Environ. Int.
30.64	104.38	Land	$PM_{2.5}$	115	1.2	4.3	-10.2	13.4	Zong et al., 2020 Environ. Int.
30.80	91.00	Land	Aerosol	7	0.5	0.8	-1.0	1.9	Lin et al., 2020 J. Geophys. Res.-Atmos.
31.26	121.57	Land	$PM_{2.5}$	47	6.6	3.4	-3.0	18.2	He et al., 2020 Sci. Total Environ.
31.29	121.50	Land	$PM_{2.5}$	108	3.6	3.9	-7.7	16.1	Zong et al., 2020 Environ. Int.
31.32	121.69	Ocean	Aerosol	1	8.3				Shi et al., 2021 Earth Planet. Sc. Lett.
31.34	121.51	Land	Aerosol	195	2.2	3.0	-8.4	13.0	Zhu et al., 2021 Atmos. Environ.
32.00	143.00	Ocean	Aerosol	1	0.9				Kamezaki et al., 2019 Atmos. Environ.
32.20	-12.60	Ocean	Aerosol	1	-3.7				Morin et al., 2009 J. Geophys. Res.
32.21	118.72	Land	$PM_{2.5}$	2	11.7				Chang et al., 2018 Atmos. Chem. Phys.
32.22	-110.92	Land	PM_{10}	49	3.8	3.1	-3.0	11.6	Riha 2014 Doctor thesis
32.27	-64.87	Ocean	Aerosol	11	-5.5	2.6	-12.1	-2.2	Gobel et al., 2013 Geophys. Res. Lett.

33.48	126.27	Land	TSP	23	11.3	4.4	1.7	21.6	Kundu et al., 2010 J. Geophys. Res.
34.70	-120.00	Land	Aerosol	38	-1.0	3.4	-7.7	10.3	Vicars et al., 2013 J. Geophys. Res.-Atmos.
35.30	139.29	Land	Aerosol	52	7.9	2.9	0.7	13.8	Mukotaka 2014 Doctor thesis
35.88	-109.43	Land	PM_{10}	58	0.2	2.3	-4.5	9.7	King 2013 Master thesis
35.97	-84.28	Land	Aerosol	6	5.6	1.5	2.9	9.	Garten 1996 Tellus B
36.26	117.10	Land	TSP	34	1.2	2.5	-6.6	7.6	Wu et al., 2021 NPJ Clim. Atmos. Sci.
37.01	129.68	Land	TSP	39	3.6	2.5	-0.6	10.1	Kim et al., 2019 Mar. Pollut. Bull.
37.10	-107.63	Land	PM_{10}	43	9.5	3.2	2.7	23.3	King 2013 Master thesis
37.20	-122.50	Land	Aerosol	15	-0.1	3.2	-6.7	6.1	Vicars et al., 2013 J. Geophys. Res.-Atmos.
37.58	127.00	Land	$PM_{2.5}$	76	7.3	2.6	3.3	16.8	Park et al., 2018 Environ. Pollut.
37.70	-11.20	Ocean	Aerosol	1	-2.2				Morin et al., 2009 J. Geophys. Res.
37.75	118.98	Land	$PM_{2.5}$	11	0.4	4.8	-8.9	14.1	Zong et al., 2018 Environ. Pollut.
37.96	124.63	Land	$PM_{2.5}$	87	6.2	4.6	-3.7	15.9	Park et al., 2018 Environ. Pollut.
37.97	114.53	Land	$PM_{2.5}$	318	6.7	4.6	-11.8	22.6	Zhang et al., 2020 Sci. Total Environ.； Luo et al., 2021 Chemosphere
38.40	120.91	Land	$PM_{2.5}$	131	7.8	5.0	-1.7	24.0	Zong et al., 2017 Environ. Sci. Technol.; Zong et al., 2019 Atmos. Oceanic Sci. Lett.
39.09	117.71	Land	PM2.5	12	7.0	4.5	-1.1	15.0	Dong et al., 2021 Sci. Total Environ.
39.11	117.17	Land	$PM_{2.5}$	146	9.2	6.7	-4.3	21.7	Tao 2017 Master thesis; Feng et al., 2019 Sci. Total Environ.; Zhang et al., 2020 Sci. Total Environ.
39.20	140.40	Land	Aerosol	242	-0.5	2.1	-4.7	5.0	Kawashima and Kurahashi 2011Atmos. Environ.; Kawashima 2014 Earozoru Kenkyu; Kawashima 2019 Tellus B
39.53	-84.73	Land	Aerosol	10	7.9	2.8	1.7	12.5	Elliott et al., 2009 J. Geophys. Res.

39.63	-83.26	Land	Aerosol	11	7.2	3.5	1.5	13.1	Elliott et al., 2009 J. Geophys. Res.
39.79	116.51	Land	PM2.5	12	6.9	4.5	-0.3	17.6	Dong et al., 2021 Sci. Total Environ.
39.93	116.33	Land	$PM_{2.5}$	112	5.5	6.1	-11.7	23.0	Zhang et al., 2020 Sci. Total Environ.
39.96	116.37	Land	$PM_{2.5}$	84	11.1	4.0	-1.0	20.3	Fan et al., 2020 J. Geophys. Res.-Atmos.
39.98	116.38	Land	$PM_{2.5}$	35	10.1	4.7	1.0	21.3	Luo et al., 2019 Sci. Total Environ.
40.00	-169.00	Ocean	Aerosol	1	-1.4				Kamezaki et al., 2019 Atmos. Environ.
40.00	152.00	Ocean	Aerosol	1	5.4				Kamezaki et al., 2019 Atmos. Environ.
40.00	116.39	Land	PM2.5	12	7.8	5.0	-1.3	17.6	Dong et al., 2021 Sci. Total Environ.
40.01	116.70	Land	$PM_{2.5}$	200	10.5	4.5	-2.3	20.9	Song et al., 2019 Environ. Pollut.; Song et al., 2019 Sci. Total Environ.; Zhang et al., 2021 Sci. Total Environ.
40.02	-105.25	Land	Aerosol	15	5.0	4.8	-1.3	18.1	Moore 1977 Atmos. Environ.
40.03	116.41	Land	$PM_{2.5}$	80	12.7	2.9	2.7	19.5	Zhang et al., Sci. Total Environ.
40.23	116.29	Land	PM2.5	12	5.8	4.6	-1.6	15.5	Dong et al., 2021 Sci. Total Environ.
40.41	116.68	Land	$PM_{2.5}$	31	7.3	5.9	-2.5	19.3	He et al., 2018 Atmos. Chem. Phys.
40.72	-77.93	Land	Aerosol	11	6.6	3.8	0.5	12.8	Elliott et al., 2009 J. Geophys. Res.
40.75	-111.88	Land	$PM_{2.5}$	3	9.5	0.7	8.5	10.5	Hall et al., 2016 J. Geophys. Res.-Atmos.
40.91	115.39	Land	PM2.5	8	2.1	5.9	-6.3	11.8	Dong et al., 2021 Sci. Total Environ.
40.92	-82.99	Land	Aerosol	11	8.4	3.3	2.3	13.7	Elliott et al., 2009 J. Geophys. Res.
41.60	-78.77	Land	Aerosol	8	8.4	3.1	3.8	12.3	Elliott et al., 2009 J. Geophys. Res.
41.83	-71.40	Land	Aerosol	6	0.6	0.5	-0.1	1.4	Blum et al., 2020 Anal. Chem.
41.84	-112.00	Land	$PM_{2.5}$	3	7.7	0.3	7.2	8.2	Hall et al., J. Geophys. Res.-Atmos.

41.94	-74.55	Land	Aerosol	8	6.0	3.3	-0.7	11.2	Elliott et al., 2009 J. Geophys. Res.
42.00	-7.87	Land	Aerosol	1	4.9				Morin et al., 2009 J. Geophys. Res.
42.40	-76.65	Land	Aerosol	10	6.8	3.5	0.2	12.6	Elliott et al., 2009 J. Geophys. Res.
43.07	141.33	Land	Aerosol	12	4.5	2.8	-0.1	11.3	Nelson et al., 2018 Atmos. Chem. Phys.
43.67	-79.41	Land	Aerosol	1	-0.9				Smirnoff et al., 2012 Rapid Commun. Mass Sp.
43.97	-74.22	Land	Aerosol	4	6.1	2.4	3.1	9.2	Elliott et al., 2009 J. Geophys. Res.
43.99	125.39	Land	$PM_{2.5}$	120	7.3	4.4	-2.7	20.0	Zhao et al., 2019 Chinese J. Anal. Chem.; Zhao et al., 2020 Atmos. Environ.
45.03	6.33	Land	Aerosol	3	-5.1	4.1	1.1	-11.3	Bourgeois et al., 2019 New Phytologist
45.10	141.20	Land	Aerosol	12	-0.4	3.2	-5.0	7.3	Nelson et al., 2018 Atmos. Chem. Phys.
45.40	-4.78	Land	Aerosol	1	6.4				Morin et al., 2009 J. Geophys. Res.
45.75	126.66	Land	$PM_{2.5}$	86	8.7	8.3	-19.1	23.1	Sun et al., 2020 Atmos. Environ
47.35	133.31	Land	$PM_{2.5}$	8	12.5	1.3	9.9	13.9	Chang et al., 2018 Atmos. Chem. Phys.
49.00	-169.00	Ocean	Aerosol	1	-1.3				Kamezaki et al., 2019 Atmos. Environ.
49.40	2.83	Land	Aerosol	1	1.0				Morin et al., 2009 J. Geophys. Res.
50.06	-112.11	Land	Aerosol	3	3.2	0.7	2.3	4.2	Savard et al., 2017 Atmos. Environ.
50.92	21.60	Land	Aerosol	68	6.6	2.7	2.7	11.9	Freyer 1991 Tellus B
52.95	1.12	Land	Aerosol	25	7.4	5.4	-2.6	19.2	Yeatman et al., 2001a Atmos. Environ.; Yeatman et al., 2001b Atmos. Environ.
53.31	-114.14	Land	Aerosol	4	6.2	1.7	4.0	8.4	Savard et al., 2017 Atmos. Environ.
53.32	-9.90	Land	Aerosol	33	-0.7	2.4	-5.5	7.3	Yeatman et al., 2001a Atmos. Environ.; Yeatman et al., 2001b Atmos. Environ.

53.45	10.35	Land	Aerosol	39	2.1	3.3	-5.1	10.5	Beyn et al., 2014 Environ. Pollut.; Beyn et al., 2015 Atmos. Environ.
53.54	-113.44	Land	Aerosol	3	5.7	1.5	4.0	7.9	Savard et al., 2017 Atmos. Environ.
53.72	-113.44	Land	Aerosol	4	2.8	4.5	-6.2	10.8	Savard et al., 2017 Atmos. Environ.
54.00	-170.00	Ocean	Aerosol	1	-0.6				Kamezaki et al., 2019 Atmos. Environ.
55.00	8.45	Land	Aerosol	39	3.2	2.8	-2.8	10.4	Beyn et al., 2015 Atmos. Environ.
57.02	-111.65	Land	$PM_{2.5}$	7	15.2	1.9	9.4	17.9	Proemse et al., 2012 Atmos. Environ.

Table S2. $\delta^{15}N$ values of dominant NO_x emission sources. Mean values with different letters differ significantly at $p<0.05$.

Sources	*n*	$\delta^{15}N$ values of NO_x emissions / ‰				References
		Mean	SD	Min.	Max.	
Coal combustion	50	14.2[a]	5.1	6.0	29.5	Refs 25, 69, 70
Biomass burning	78	0.28[b]	2.7	-8.0	12.0	Refs 33-36
Oil combustion	182	-7.1[c]	4.2	-20.9	-5.8	Refs 25-31, 69, 71
Microbial N cycle	140	-37.0[d]	13.5	-61.8	-8.3	Refs 63-68

Table S3. The data sources and values of parameters used for calculating ambient $^{15}\Delta_{i\text{-}NOx \rightarrow p\text{-}NO3\text{-}}$ values. In scenario 1, mean values of regional $\delta^{15}N_{NOx}$ and f_{NO2} values and simultaneously observed values of ambient C_{NO2}, C_{HNO3}, $C_{p\text{-}NO3\text{-}}$, $\delta^{15}N_{HNO3}$, and $\delta^{15}N_{p\text{-}NO3\text{-}}$ were used. In scenario 2, non-synchronously observed values of ambient f_{NO2}, C_{NO2}, C_{HNO3}, $C_{p\text{-}NO3\text{-}}$, $\delta^{15}N_{NOx}$, $\delta^{15}N_{HNO3}$, and $\delta^{15}N_{p\text{-}NO3\text{-}}$ were used. (a) data were cited from Supplementary Texts S3 and S4; (b) data were cited from Supplementary Text S5; (c) data were cited from Supplementary Text S6; (d) data were cited from Supplementary Texts S2, S7, and S8.

Observations	Site, Country/Region	NO_2 (μg N m^{-3})			f_{NO2} (%) (b)			HNO_3 (μg N m^{-3})			p-NO_3^- (μg N m^{-3})		
		Mea	SD	*n*	Mea	SD	*n*	Mean	SD	*n*	Mean	SD	*n*
Scenario 1	Jülich, Germany	1.0	0.4	19	62.0	15.	91	0.4	0.1	12	1.2	0.2	12
	Pennsylvania, USA	0.4	0.2	6	74.0	12.	74	0.2	0.0	11	0.1	0.1	11
	Rishiri, Japan	0.3	0.1	12	64.0	17.	72	0.1	0.0	12	0.2	0.1	12
	Sapporo, Japan	2.6	0.8	11	64.0	17.	72	0.1	0.1	12	0.3	0.1	12
	Alberta, Canada	0.9	0.1	7	74.0	12.	74	0.5	0.2	15	0.3	0.3	15
Scenario 2	East Asia	8.9	1.1	19	64.0	17.	72	0.2	0.0	532	0.3	0.1	546
	Europe	1.9	0.1	20	62.0	15.	91	0.2	0.1	200	0.5	0.3	200
	North America	7.3	1.0	16	74.0	12.	74	0.6	0.1	420	0.2	0.1	421

(continued)

Observations	Site, Country/ Region	$\delta^{15}N_{NOx}$ (‰) (c)			$\delta^{15}N_{HNO3}$ (‰) (d)			$\delta^{15}N_{p\text{-}NO3\text{-}}$ (‰) (d)		
		Mean	SD	*n*	Mean	SD	*n*	Mean	SD	*n*
Scenario 1	Jülich, Germany	-4.9	4.4	84	-2.5	0.6	12	7.3	2.6	12
	Pennsylvania, USA	-13.8	6.6	325	4.3	2.8	11	7.0	3.6	11
	Rishiri, Japan	-9.2	2.6	39	-0.7	3.3	13	1.8	1.7	12
	Sapporo, Japan	-9.2	2.6	39	2.7	1.4	13	6.2	3.1	13
	Alberta, Canada	-13.8	6.6	325	-0.9	3.3	5	5.3	2.7	5
Scenario 2	East Asia	-9.2	2.6	39	-1.0	4.9	49	5.4	5.1	3493
	Europe	-4.9	4.4	84	-2.5	0.9	13	3.9	3.8	208
	North America	-13.8	6.6	325	0.4	3.9	287	5.3	4.2	279

SI References

1. http://isiknowledge.com
2. http://scholar.google.com.hk
3. http://xueshu.baidu.com
4. Rohatgi A. WebPlotDigitalizer: HTML5 based online tool to extract numerical data from plot images. Version 4.2. https://automeris.io/WebPlotDigitizer/index.html (accessed on April 2019) (2019).
5. Tan JN, Fu JS and Dentener F *et al*. Multi-model study of HTAP II on sulfur and nitrogen deposition. *Atmos Chem Phys* 2018; **18**: 6847–6866.
6. Altieri KE, Fawceet SE and Hastings MG. Reactive nitrogen cycling in the atmosphere and ocean. *Annu Rev Earth Planet Sci* 2021; **49**: 523–550.
7. Jickells TD, Buitenhuis E and Altieri K *et al*. A reevaluation of the magnitude and impacts of anthropogenic atmospheric nitrogen inputs on the ocean. *Global Biogeochem Cy* 2017; **31**: 289–305.
8. Dentener F, Drevet J and Lamarque JF *et al*. Nitrogen and sulfur deposition on regional and global scales: A multimodel evaluation. *Global Biogeochem Cy* 2006b; **20**: 21.
9. Duce RA, Laroche J and Altieri K *et al*. Impacts of atmospheric anthropogenic nitrogen on the open ocean. *Science* 2008; **320**: 893–897.
10. Vet R, Artz RS and Carou S *et al*. A global assessment of precipitation chemistry and deposition of sulfur, nitrogen, sea salt, base cations, organic acids, acidity and pH, and phosphorus. *Atmos Environ* 2014; **93**: 3–100.
11. Parrish DD, Buhr MP and Trainer M *et al*. The total reactive oxidized nitrogen levels and the partitioning between the individual species at six rural sites in Eastern North America. *J Geophys Res* 1993; **98**: 2927–2939
12. Hayden KL, Anlauf KG and Hastie DR *et al*. Partitioning of reactive atmospheric nitrogen oxides at an elevated site in southern Quebec, Canada. *J Geophys Res* 2003; **108**: 4603.
13. Zhang LM, Wiebe A and Vet R. *et al*. Measurements of reactive oxidized nitrogen at eight Canadian rural sites. *Atmos Environ* 2008; **42**: 8065–8078.
14. Zhang LM, Vet R and O'Brien JM *et al*. Dry deposition of individual nitrogen species at eight Canadian rural sites. *J Geophys Res* 2009; **114**: D02301.

15. Fang Y, Fiore AM and Horowitz LW *et al*. Sensitivity of the NO_y budget over the United States to anthropogenic and lightning NO_x in summer. *J Geophys Res* 2010; **115**: D18312.

16. Li JJ, Mao JQ and Fiore AM *et al*. Decadal changes in summertime reactive oxidized nitrogen and surface ozone over the Southeast United States. *Atmos Chem Phys* 2018; **18**: 2341–2361.

17. Ninnerman M, Marto J and Shaw S *et al*. Reactive oxidized nitrogen speciation and partitioning in urban and rural New York State. *J Air Waste Manage* 2021; **71**: 348–365.

18. Theys N, Volkamer R and Müller JF *et al*. Global nitrous acid emissions and levels of regional oxidants enhanced by wildfires. *Nat Geosci* 2020; **13**: 1–6.

19. Stutz J, Alicke B and Ackermann R *et al*. Relative humidity dependence of HONO chemistry in urban areas. *J Geophys Res* 2004; **109**: D03307.

20. Li X, Rohrer F and Hofzumahaus A *et al*. Missing gas-phase source of HONO inferred from zeppelin measurements in the troposphere. *Science* 2014; **344**: 292–296.

21. Walters WW and Michalski G. Theoretical calculation of nitrogen isotope equilibrium exchange fractionation factors for various NO_y molecules. *Geochim Cosmochim Ac* 2015; **164**: 284–297.

22. Freyer H, Kley D and Vozl-Thomas A *et al*. On the interaction of isotopic exchange processes with photochemical reactions in atmospheric oxides of nitrogen. *J Geophys Res-Atmos* 1993; **98**: 14791–14796.

23. Liu XY, Yin YM and Song W. Nitrogen isotope differences between major atmospheric NO_y species: Implications for transformation and deposition processes. *Environ Sci Tech Let* 2020; **7**: 227–233.

24. Song W, Liu XY and Liu CQ. New constraints on isotopic effects and major sources of nitrate in atmospheric particulates by combining $\delta^{15}N$ and $\Delta^{17}O$ signatures. *J Geophys Res-Atmos* 2021a; https://doi.org/10.1029/2020JD034168.

25. Wang X, Ti CP and Luo YX *et al*. Determination of ^{15}N natural abundance in nitrogen oxides from major anthropogenic emission sources. *Acta Pedologica Sinica* 2016; **53**: 1552–1562 (2016). (In Chinese with English Abstract)

26. Zong Z, Sun ZY and Xiao LL *et al*. Insight into the variability of the nitrogen isotope composition of vehicular NO_x in China. *Environ Sci Technol* 2020; **54**: 14246–14253.

27. Moore H. The isotopic composition of ammonia, nitrogen dioxide and nitrate in the atmosphere. *Atmos Environ* 1977; **11**: 1239–1243.

28. Fibiger DL, Hastings MG and Lew AF *et al*. Collection of NO and NO_2 for isotopic analysis of NO_x emissions. *Anal Chem* 2014; **86**: 12115–12121.

29. Walters WW, Thap BD and Fang H *et al*. Nitrogen isotope composition of thermally produced NO_x from various fossil-fuel combustion sources. *Environ Sci Technol* 2015; **49**: 11363–11371.

30. Wojtal PK, Miller DJ and O'Connor M *et al*. Automated, high-resolution mobile collection system for the nitrogen isotopic analysis of NO_x. *J Vis Exp* 2016; **118**: e54962.

31. Miller DJ, Wojtal PK and Clark SC *et al*. Vehicle NO_x emission plume isotopic signatures: Spatial variability across the eastern United States. *J Geophys Res-Atmos* 2017; **122**: 4698–4717.

32. Craine JM, Elmore AJ and Wang LX *et al*. Isotopic evidence for oligotrophication of terrestrial ecosystems. *Nat Ecol Evol* 2018; **2**: 1734–1744.

33. Fibiger DL and Hastings MG. First measurements of the nitrogen isotopic composition of NO_x from biomass burning. *Environ Sci Technol* 2016; **50**: 11569–11574.

34. Chai JJ, Miller DJ and Scheuer E *et al*. Isotopic characterization of nitrogen oxides (NO_x), nitrous acid (HONO), and nitrate (NO_3^-(p)) from laboratory biomass burning during FIREX. *Atmos Meas Tech* 2019; **12**: 6303–6317.

35. Yin YM. Emissions and isotopic characteristics of particulate matters and nitrogen oxides from biomass burning in North China. Master thesis, Tianjin University. 2017. (In Chinese with English Abstract)

36. Shi YS, Tian P and Jin ZF *et al*. Stable nitrogen isotope composition of NO_x of biomass burning in China. *Sci Total Environ* 2021; **803**: 149857.

37. Hietz P, Turmer BL and Wanek W *et al*. Long-term change in the nitrogen cycle of tropical forests. *Science* 2011; **334**: 664–666.

38. Mason RE, Craine JM and Lany NK *et al*. Evidence, causes, and consequences of declining nitrogen availability in terrestrial ecosystems. *Science* 2022; **376**: 261.

39. Parnell AC, Inger R and Bearhop S *et al*. Source partitioning using stable isotopes: coping with too much variation. *PLoS ONE* 2010; **5**: e9672.

40. Evans JSBT, Handley SJ and Perham N *et al*. Frequency versus probability formats in statistical word problems. *Cognition* 2000; **77**: 197–213.

41. Moore JW and Semmens BX. Incorporating uncertainty and prior information into stable isotope mixing models. *Ecol Lett* 2008; **11**: 470–480.

42. Liu XY, Koba K and Koyama *et al*. Nitrate is an important nitrogen source for Arctic tundra plants. *PNAS* 2018; **115**: 3398–3403.

43. Liu XY, Xiao HW and Xiao HY *et al*. Stable isotope analyses of precipitation nitrogen sources in Guiyang, Southwestern China. *Environ Pollut* 2017; **230**: 486–494.

44. Zong Z, Wang XP and Tian CG *et al*. First assessment of NO_x sources at a regional background site in North China using isotopic analysis linked with modeling. *Environ Sci Technol* 2017; **51**: 5923–5931.

45. Song W, Liu XY and Wang YL *et al*. Nitrogen isotope differences between atmospheric nitrate and corresponding nitrogen oxides: A new constraint using oxygen isotopes. *Sci Total Environ* 2020; **701**: 134515.

46. Song W, Liu XY and Hu CC *et al*. Important contributions of non-fossil fuel nitrogen oxides emissions. *Nat Commun* 2021b; **12**: 243.

47. Fowler D, Coyle M and Skiba U *et al*. The global nitrogen cycle in the twenty-first century. *Phil Trans R Soc B* 2013; **368**: 20130164.

48. Jaeglé L, Steinberger L and Martin RV *et al*. Global partitioning of NO_x sources using satellite observations: Relative roles of fossil fuel combustion, biomass burning and soil emissions. *Faraday discussions* 2005; **130**: 407–423.

49. IPCC: Climate Change 2013: Carbon and Other Biogeochemical Cycles. Contribution of Working Group I to the Fifth Assessment Report of the Intergovernmental Panel on Climate Change, edited by: Ciais P, Sabine C and Bala G *et al*. Cambridge University Press. 2013, 465-570.

50. Dentener F, Stevenson D and Ellingsen K *et al*. The global atmospheric environment for the next generation. *Environ Sci Technol* 2006a; **40**: 3586–3594.

51. Vuuren DPV, Bouwman LF and Smith SJ *et al*. Global projections for anthropogenic reactive nitrogen emissions to the atmosphere: an assessment of scenarios in the scientific literature. *Curr Opin Env Sust* 2011; **3**: 359–369.

52. Corbett JJ and Koehler HW. Updated emissions from ocean shipping. *J Geophys Res-Atmos* 2003; **108**: 4650.

53. Eyring V, Köhler HW and Aardenne JV *et al*. Emissions from international shipping: 1. The last 50 years. *J Geophys Res-Atmos* 2005; **110**: D17305.

54. Eyring V, Isaksen ISA and Berntsen T *et al*. Transport impacts on atmosphere and climate: Shipping. *Atmos Environ* 2010; **44**: 4735–4771.

55. Holmes CD, Prather M and Vinken GCM. The climate impact of ship NO_x emissions: an improved estimate accounting for plume chemistry. *Atmos Chem Phys* 2014; **14**: 6801–6812.

56. Yan F, Winijkul E and Streets DG *et al*. Global emission projections for the transportation sector using dynamic technology modeling. *Atmos Chem Phys* 2014; **14**: 5709–5733.

57. Johansson L, Jalkanen JP and Kukkonen J. Global assessment of shipping emissions in 2015 on a high spatial and temporal resolution. *Atmos Environ* 2017; **167**: 403–415.

58. Jonson JE, Gauss M and Schulz M *et al*. Effects of global ship emissions on European air pollution levels. *Atmos Chem Phys* 2020; **20**: 11399–11422.

59. Lamarque JF, Shindell DT and Josse B *et al*. The Atmospheric Chemistry and Climate Model Intercomparison Project (ACCMIP): Overview and description of models, simulations and climate diagnostics. *Geosci Model Dev* 2013a; **6**: 179–206.

60. Lamarque JF, Dentener F and Mcconnell J *et al*. Multi-model mean nitrogen and sulfur deposition from the Atmospheric Chemistry and Climate Model Intercomparison Project (ACCMIP): Evaluation of historical and projected future changes. *Atmos Chem Phys* 2013b; **13**: 7997–8018.

61. Kanakidou M, Myriokefalitakis S and Daskalakis N *et al*. Past, present, and future atmospheric nitrogen deposition. *J Atmos Sci* 2016; **73**: 2039–2047.

62. Geddes JA and Martin RV. Global deposition of total reactive nitrogen oxides from 1996 to 2014 constrained with satellite observations of NO_2 columns. *Atmos Chem Phys* 2017; **17**: 10071–10091.

63. Li DJ and Wang XM. Nitrogen isotopic signature of soil-released nitric oxide (NO) after fertilizer application. *Atmos Environ* 2008; **42**: 4747–4754.

64. Su CX, Kang RH and Zhu WX *et al*. $\delta^{15}N$ of nitric oxide produced under aerobic or anaerobic conditions from seven soils and their associated N isotope fractionations. *J Geophys Res-Biogeo* 2020; **125**: e2020JG005705.

65. Felix JD and Elliott EM. The isotopic composition of passively collected nitrogen dioxide emissions: Vehicle, soil and livestock source signatures. *Atmos Environ* 2014; **92**: 359–366.

66. Yu ZJ and Elliott EM. Novel method for nitrogen isotopic analysis of soil-emitted nitric oxide. *Environ Sci Technol* 2017; **51**: 6268–6278.

67. Felix JD and Elliott EM. The agricultural history of human-nitrogen interactions as recorded in ice core $\delta^{15}N\text{-}NO_3^-$. *Geophys Res Lett* 2013; **40**: 1642–1646.

68. Miller DJ, Chai JJ and Guo F *et al*. Isotopic composition of in situ soil NO_x emissions in manure-fertilized cropland. *Geophys Res Lett* 2018; **45**: 12058–12066.

69. Heaton THE. $^{15}N/^{14}N$ ratios of NO_x from vehicle engines and coal-fired power station. *Tellus B* 1990; **42**: 304–307.

70. Felix JD, Elliott EM and Shaw SL. The isotopic composition of coal-fired power plant NO_x: The influence of emission controls and implications for global emission inventories. *Environ Sci Technol* 2012; **46**: 3528–3535.

71. Walters WW, Goodwin SR and Michalski G. Nitrogen stable isotope composition ($\delta^{15}N$) of vehicle-emitted NO_x. *Environ Sci Technol* 2015a; **49**: 2278–2285.

Supplementary Text

Text S1. Publications of the global NO_x production from lighting

1. Lawrence MG, Chameides WL and Kasibhatla PS *et al*. Lightning and atmospheric chemistry: The rate of atmospheric NO production. In H. Volland (Ed.), Handbook of atmospheric electrodynamics. CRC Press, 1995, 189-202.

2. Levy H II, Moxim WJ and Kasibhatla PS. A global three-dimensional time-dependent lightning source of tropospheric NO_x. *J Geophys Res* 1996; **101**: 22911–22922.

3. Lee DS, Köhler I and Grobler E *et al*. Estimations of global NO_x emissions and their uncertainties. *Atmos Environ* 1997; **31**: 1735–1749.

4. Huntrieser H, Schlager H and Feigl C *et al*. Transport and production of NO_x in electrified thunderstorms: Survey of previous studies and new observations at mid-latitudes. *J Geophys Res* 1998; **103**: 247–328.

5. WMO, Scientific assessment of ozone depletion (1998). World Meteorological Organization, Global Ozone Research and Monitoring Project- Report No. 44, Geneva, Switzerland, 1999.

6. Bradshaw J, Davis D and Grodzinsky G *et al*. Observed distributions of nitrogen oxides in the remote free troposphere from the NASA global tropospheric experiment programs. *Rev Geophys* 2000; **38**: 61–116.

7. IPCC: Climate Change 2001: Synthesis report. Contribution of Working Groups I, II, and III to the Third Assessment Report of the Intergovernmental Panel on Climate Change. edited by: Watson RT, Albritton DL and Barker T *et al*. Cambridge University Press. 2001, 1-397.

8. Tie XX, Zhang RY and Brassuer GP *et al*. Global NO_x production by lightning. *J Atmos Chem* 2002; **43**: 61–74.

9. Galloway JN, Dentener FJ and Capone DG *et al*. Nitrogen cycles: Past, present, and future. *Biogeochemistry* 2004; **70**: 153–226.

10. Beirle S,Platt U and von Glasow R *et al*. Estimate of nitrogen oxide emissions from shipping by satellite remote sensing. *Geophys Res Lett* 2004; **31**: L18102.

11. Boersma KF, Eskes HJ and Meijer EW *et al*. Estimates of lightning NO_x production from GOME satellite observations. *J Geophys Res* 2005; **106**: 27701–27710.

12. Schumann U and Huntrieser H. The global lightning-induced nitrogen oxides source. *Atmos Chem Phys* 2007; **7**: 3823–3907.

13. Huntrieser H, Schlager H and Lichtenstern M *et al*. NO_x production by lightning in Hector: First airborne measurements during SCOUT-O3/ACTIVE. *Atmos Chem Phys* 2009; **9**: 8377–8412.

14. Jourdain L, Sulawik SS and Worden HM *et al*. Lightning NO_x emissions over the USA constrained by TES ozone observations and the GEOS-Chem model. *Atmos Chem Phys* 2010; **10**: 107–119.

15. Martini M, Allen DJ and Pickering KE *et al*. The impact of North American anthropogenic emissions and lightning on longrange transport of trace gases and their export from the continent during summers 2002 and 2004. *J Geophys Res-Atmos* 2011; **116**: D07305.

16. Murray LT, Jacob DJ and Logan JA *et al*. Optimized regional and inter-annual variability of lightning in a global chemical transport model constrained by LIS/OTD satellite data. *J Geophys Res Atmos* 2012; **117**: D20307.

17. Miyazaki K, Eskes HJ and Sudo K *et al*. Global lightning NO_x production estimated by an assimilation of multiple satellite data sets. *Atmos Chem Phys* 2014; **14**: 3277–3305.

18. Liaskos CE, Allen DJ and Pickering KE. Sensitivity of tropical tropospheric composition to lightning NO_x production as determined by replay simulations with GEOS-5. *J Geophys Res-Atmos* 2015; **120**: 8512–8534.

19. Nault BA, Laughner JL and Wooldridge PJ *et al*. Lightning NO_x emissions: Reconciling measured and modeled estimates with updated NO_x chemistry. *Geophys Res Lett* 2017; **44**: 9479–9488.

20. Marais EA, Jacob DJ and Choi S *et al*. Nitrogen oxides in the global upper troposphere: interpreting cloud-sliced NO_2 observations from the OMI satellite instrument. *Atmos Chem Phys* 2018; **18**: 17017–17027.

21. Verma S, Yadava PK and Lal DM *et al*. Role of Lightning NO_x in Ozone Formation: A Review. *Pure Appl Geophys* 2021; **178**: 1425–1443.

Text S2. Publications of $\delta^{15}N_{p\text{-}NO3\text{-}}$ observations.

1. Moore H. The isotopic composition of ammonia, nitrogen dioxide and nitrate in the atmosphere. *Atmos Environ* 1997; **11**: 1239–1243.

2. Heaton THE. $^{15}N/^{14}N$ ratios of nitrate and ammonium in rain at Pretoria, South Africa. *Atmos Environ* 1987; **21**: 843–852.

3. Freyer HD. Seasonal variation of $^{15}N/^{14}N$ ratios in atmospheric nitrate species. *Tellus B* 1991; **43**: 30–44.

4. Charles T and Garten JR. Stable nitrogen isotope ratios in wet and dry nitrate deposition collected with an artificial tree. *Tellus B* 1996; **48**: 60–64.

5. Yeatman SG, Spokes LJ and Dennis PF *et al*. Can the study of nitrogen isotopic composition in size-segregated aerosol nitrate and ammonium be used to investigate atmospheric processing mechanisms? *Atmos Environ* 2001; **35**: 1337–1345.

6. Yeatman SG, Spokes LJ and Dennis PF *et al*. Comparisons of aerosol nitrogen isotopic composition at two polluted coastal sites. *Atmos Environ* 2001; **35**: 1307–1320.

7. Baker AR, Weston K and Kelly SD *et al*. Dry and wet deposition of nutrients from the tropical Atlantic atmosphere: Links to primary productivity and nitrogen fixation. *Deep Sea Res* 2007; **54**: 1704–1720.

8. Elliott EM, Kendal C and Boyer EW *et al*. Dual nitrate isotopes in dry deposition: Utility for partitioning NO_x source contributions to landscape nitrogen deposition. *J Geophys Res-Biogeo* 2009; **114**: G04020.

9. Morin S, Savarino J and Frey MM *et al*. Comprehensive isotopic composition of atmospheric nitrate in the atlantic ocean boundary layer from 65°S to 79°N. *J Geophys Res-Atmos* 2009; **114**: D05303.

10. Kundu S, Kawamura K and Lee M. Seasonal variation of the concentrations of nitrogenous species and their nitrogen isotopic ratios in aerosols at Gosan, Jeju Island: Implications for atmospheric processing and source changes of aerosols. *J Geophys Res-Atmos* 2010; **115**: D20305.

11. Wankel SD, Chen Y and Kendall C *et al*. Sources of aerosol nitrate to the gulf of Aqaba: evidence from $\delta^{15}N$ and $\delta^{18}O$ of nitrate and trace metal chemistry. *Mar Chem* 2010; **120**: 90–99.

12. Kawashima T and Kurahashi T. Inorganic ion and nitrogen isotopic compositions of atmospheric aerosols at Yurihonjo, Japan: implications for nitrogen sources. *Atmos Environ* 2011; **45**: 6309–6313.

13. Proemse BC, Mayer B and Chow JC *et al*. Isotopic characterization of nitrate, ammonium and sulfate in stack $PM_{2.5}$ emissions in the Athabasca oil sands region, Alberta, Canada. *Atmos Environ* 2012; **60**: 555–563.

14. Smirnoff A, Savard MM and Vet R *et al*. Nitrogen and triple oxygen isotopes in near-road air samples using chemical conversion and thermal decomposition. *Rapid Commun Mass Sp* 2012; **26**: 2791–2804.

15. Gobel AR, Altieri KE and Peters AJ *et al*. Insights into anthropogenic nitrogen deposition to the North Atlantic investigated using the isotopic composition of aerosol and rainwater nitrate. *Geophys Res Lett* 2013; **40**: 5977–5982.

16. King MZ. Evaluating NO_x sources and oxidation pathways impacting aerosol production on the Southern Ute Indian Reservation and Navajo Nation using geochemical isotopic analysis. Master thesis, Purdue University. 2013.

17. Savarino J, Morin S and Erbland J *et al*. Isotopic composition of atmospheric nitrate in a tropical marine boundary layer. *PNAS* 2013; **110**: 17668−17673 (2013).

18. Vicars WC, Morin S and Savarino J *et al*. Spatial and diurnal variability in reactive nitrogen oxide chemistry as reflected in the isotopic composition of atmospheric nitrate: results from the Calnex 2010 field study. *J Geophys Res-Atmos* 2013; **118**: 10567–10588.

19. Mukotaka A. A study of nitrogen oxides dynamics between urban atmosphere and the phyllosphere using triple oxygen isotopes. Doctoral thesis, Tokyo Institute of Technology. 2014.

20. Beyn F, Matthias V and Dahnke K. Changes in atmospheric nitrate deposition in Germany-an isotopic perspective. *Environ Pollut* 2014; **194**: 1–10.

21. Kawashima H. Formation mechanism and source apportionment of ammonium and nitrate ions in aerosol using nitrogen isotopes. *Earozoru Kenkyu* 2014; **29**: 110–116.

22. Riha KM. The use of stable isotopes to constrain the nitrogen cycle. Doctoral thesis, Purdue University. 2013.

23. Wang F, Michalski G and Seo JH *et al*. Geochemical, isotopic, and mineralogical constraints on atmospheric deposition in the hyper-arid Atacama Desert, Chile. *Geochim Cosmochim Ac* 2014; **135**: 29–48.

24. Yang JYT., Hsu SC and Dai MH *et al*. Isotopic composition of water-soluble nitrate in bulk atmospheric deposition at Dongsha Island: sources and implications of external N supply to the northern South China Sea. *Biogeosciences* 2014; **11**: 1833–1846.

25. Beyn F, Matthias V and Aulinger A *et al*. Do N-isotopes in atmospheric nitrate deposition reflect air pollution levels? *Atmos Environ* 2015; **107**: 281–288.

26. Xiao HW, Xie LH and Long AM *et al*. Use of isotopic compositions of nitrate in TSP to identify sources and chemistry in South China Sea. *Atmos Environ* 2015; **109**: 70–78.

27. Hall SJ, Ogata EM and Weintraub SR *et al*. Convergence in nitrogen deposition and cryptic isotopic variation across urban and agricultural valleys in northern Utah. *J Geophys Res-Biogeo* 2016; **121**: 2340–2355.

28. Guha T, Lin CT and Bhattacharya SK *et al*. Isotopic ratios of nitrate in aerosol samples from Mt. Lulin, a high-altitude station in Central Taiwan. *Atmos Environ* 2017; **154**: 53–69.

29. Savard MM, Cole A and Smirnoff A *et al*. $\delta^{15}N$ values of atmospheric N species simultaneously collected using sector-based samplers distant from sources – isotopic inheritance and fractionation. *Atmos Environ* 2017; **162**: 11–22.

30. Zong Z, Wang XP and Tian CG *et al*. First assessment of NO_x sources at a regional background site in North China using isotopic analysis linked with modeling. Environ Sci Technol 2017; **51**: 5923–5931.

31. Li QK. Trace the sources of urban aerosol nitrate emitted from vehicle exhaust by using stable nitrogen and oxygen isotopes. Master thesis, University of Chinese Academy of Sciences. 2017.

32. Tao YL. Study on characteristics and sources of $PM_{2.5}$ pollution in Tianjin urban area based on stable isotope technique. Master thesis, Tianjin University. 2017.

33. Chang YH, Zhang YL and Tian CG *et al*. Nitrogen isotope fractionation during gas-to-particle conversion of NO_x to NO_3^- in the atmosphere-implications for isotope-based NO_x source apportionment. *Atmos Chem Phys* 2018; **18**: 11647–11661.

34. He PZ, Xie ZQ and Chi XY *et al*. Atmospheric $\Delta^{17}O(NO_3^-)$ reveals nocturnal chemistry dominates nitrate production in Beijing haze. *Atmos Chem Phys* 2018; **18**: 14465–14476.

35. Nelson DM, Tsunogai U and Ding D *et al*. Triple oxygen isotopes indicate urbanization affects sources of nitrate in wet and dry atmospheric deposition. *Atmos Chem Phys* 2018; **18**: 6381–6392.

36. Park YM, Park KS and Kim H *et al*. Characterizing isotopic compositions of TC-C, NO_3^--N, and NH_4^+-N in $PM_{2.5}$ in South Korea: impact of China's winter heating. *Environ Pollut* 2017; **233**: 735–744.

37. Zong Z, Tan Y and Wang XP *et al*. Assessment and quantification of NO_x sources at a regional background site in north china: comparative results from a Bayesian isotopic mixing model and a positive matrix factorization model. *Environ Pollut* 2018; **242**: 1379–1386.

38. Bourgeois I, Clement JC and Caillon N *et al*. Foliar uptake of atmospheric nitrate by two dominant subalpine plants: insights from in situ triple-isotope analysis. *New Phytol* 2019; **223**: 1687–1689.

39. Feng XQ, Li QK and Tao YL *et al*. Impact of Coal Replacing Project on atmospheric fine aerosol nitrate loading and formation pathways in urban Tianjin: Insights from chemical composition and ^{15}N and ^{18}O isotope. *Sci Total Environ* 2020; **15**: 134797.

40. Kamezaki K, Hattori S and Iwamoto Y *et al*. Tracing the sources and formation pathways of atmospheric particulate nitrate over the Pacific Ocean using stable isotopes. *Atmos Environ* 2019; **209**: 152–166.

41. Kawashima H. Seasonal trends of the stable nitrogen isotope ratio in particulate nitrogen compounds and their gaseous precursors in Akita, Japan. *Tellus B* 2019; **71**: 1627846.

42. Kim H, Park GH and Lee SE *et al*. Stable isotope ratio of atmospheric and seawater nitrate in the East Sea in the northwestern Pacific Ocean. *Mar Pollut Bull* 2019; **149**: 110610.

43. Luo L, Wu YF and Xiao HY *et al*. Origins of aerosol nitrate in Beijing during late winter through spring. *Sci Total Environ* 2019; **653**: 776–782.

44. Song W, Wang YL and Yang W *et al*. Isotopic evaluation on relative contributions of major NO_x sources to nitrate of $PM_{2.5}$ in Beijing. *Environ Pollut* 2019; **248**: 183–190.

45. Zhao ZY, Cao F and Zhang WQ *et al*. Determination of stable nitrogen and oxygen isotope ratios in atmospheric aerosol nitrates. *Chinese J Anal Chem* 2019; **47**: 907–915.

46. Zong Z, Sun ZY and Tan Y *et al*. Impact of an accidental explosion in Tianjin Port on enhanced atmospheric nitrogen deposition over the Bohai Sea inferred from aerosol nitrate dual isotopes. *Atmos Ocean Sci Lett* 2019; **13**: 195-201.

47. He PZ, Xie ZQ and Yu XW *et al*. The observation of isotopic compositions of atmospheric nitrate in Shanghai China and its implication for reactive nitrogen chemistry. *Sci Total Environ* 2020; **714**: 136727.

48. Luo L, Pan YY and Zhu RG *et al*. Assessment of the seasonal cycle of nitrate in $PM_{2.5}$ using chemical compositions and stable nitrogen and oxygen isotopes at Nanchang, China. *Atmos Environ* 2020; **225**: 117371.

49. Song W, Liu XY and Wang YL *et al*. Nitrogen isotope differences between atmospheric nitrate and corresponding nitrogen oxides: A new constraint using oxygen isotopes. *Sci Total Environ* 2020; **701**: 134515.

50. Zhang ZY, Zheng NJ and Zhang D *et al*, Rayleigh based concept to track NO_x emission sources in urban areas of China. *Sci Total Environ 2020*; **704**: 135362.

51. Zong Z, Tan Y and Wang X *et al*. Dual-modelling-based source apportionment of NO_x in five Chinese megacities: Providing the isotopic footprint from 2013 to 2014. *Environ Int* 2020; **137**: 105592.

52. Blum DE, Walters WW and Hastings MG. Speciated collection of nitric acid and fine particulate nitrate for nitrogen and oxygen stable isotope determination. *Anal Chem* 2020; **92**: 16079–16088.

53. Fan MY, Zhang YL and Lin YC *et al*. Changes of emission sources to nitrate aerosols in Beijing after the clean air actions: Evidence from dual isotope compositions. *J Geophys Res-Atmos* 2020; **125**: e2019JD031998.

54. Lin M, Hattori S and Wang K *et al*. A complete isotope ($\delta^{15}N$, $\delta^{18}O$, $\Delta^{17}O$) investigation of atmospherically deposited nitrate in glacial-hydrologic systems across the Third Pole region. *J Geophys Res Atmos* 2020; **125**: e2019JD031878.

55. Su T, Li J and Tian CG *et al*. Source and formation of fine particulate nitrate in South China: Constrained by isotopic modeling and online trace gas analysis. *Atmos Environ* 2020; **231**: 117563.

56. Sun XZ, Zong Z and Wang K *et al*. The importance of coal combustion and heterogeneous reaction for atmospheric nitrate pollution in a cold metropolis in China: Insights from isotope fractionation and Bayesian mixing model. *Atmos Environ* 2020; **243**: 117730.

57. Wang K, Hattori S and Kang SC *et al*. Isotopic constraints on the formation pathways and sources of atmospheric nitrate in the Mt. Everest region. *Environ Pollut* 2020; **267**: 115274.

58. Xiao HW, Zhu RG and Pan YY *et al*. Differentiation between nitrate aerosol formation pathways in a southeast Chinese city by dual isotope and modeling studies. *J Geophys Res-Atmos* 2020; **125**: e2020JD032604.

59. Zhang ZY, Guan H and Luo L *et al*. Sources and transformation of nitrate aerosol in winter 2017–2018 of megacity Beijing: Insights from an alternative approach. *Atmos Environ* 2020; **241**: 117842.

60. Zhao ZY, Cao F and Fan MY *et al*. Coal and biomass burning as major emissions of NO_x in Northeast China: Implication from dual isotopes analysis of fine nitrate aerosols. *Atmos Environ* 2020; **242**: 117762.

61. Fang Y. Chemical characteristics and tracer study of nitrate sources using stable nitrogen and oxygen isotopes in atmosphere aerosols over the East China sea island. Master thesis, Nanjing University of Information Science and Technology. 2020. (In Chinese with English Abstract).

62. Guo W, Luo L and Zhang ZY *et al*. The use of stable oxygen and nitrogen isotopic signatures to reveal variations in the nitrate formation pathways and sources in different seasons and regions in China. *Environ Res* 2021; **201:** 111537.

63. Jin ZF, Qian LJ and Shi YS *et al*. Quantifying major NO_x sources of aerosol nitrate in Hangzhou, China, by using stable isotopes and a Bayesian isotope mixing model. *Atmos Environ* 2021; **244**: 117979.

64. Li JHY, Davy P and Harvey M *et al*. Nitrogen isotopes in nitrate aerosols collected in the remote marine boundary layer: Implications for nitrogen isotopic fractionations among atmospheric reactive nitrogen species. *Atmos Environ* 2021; **245**: 118028.

65. Li QK, Li XD and Yang Z *et al*. Diurnal and seasonal variations in water-soluble inorganic ions and nitrate dual isotopes of $PM_{2.5}$: Implications for source apportionment and formation processes of urban aerosol nitrate. *Atmos Res* 2021; **248**: 105197.

66. Luo L, Zhu RG and Song CB *et al*. Changes in nitrate accumulation mechanisms as $PM_{2.5}$ levels increase on the North China Plain: A perspective from the dual isotopic compositions of nitrate. *Chemosphere* 2021; **263**: 127915.

67. Shi GT, Ma HM and Zhu ZY *et al*. Using stable isotopes to distinguish atmospheric nitrate production and its contribution to the surface ocean across hemispheres. *Earth Planet Sci Lett* 2021; **564**: 116914.

68. Wu LB, Yue SY and Shi ZB *et al*. Source forensics of inorganic and organic nitrogen using $\delta^{15}N$ for tropospheric aerosols over Mt. Tai. *npj Clim Atmos Sci* 2021; **4**: 8.

69. Zhang ZY, Guan H and Xiao HW *et al*. Oxidation and sources of atmospheric NO_x during winter in Beijing based on $\delta^{15}N$-$\delta^{18}O$ space of particulate nitrate. *Environ Pollut* 2021; **276**: 116708.

70. Zhang ZY, Cao L and Liang Y *et al*. Importance of NO_3 radical in particulate nitrate formation in a southeast Chinese urban city: New constraints by $\delta^{15}N$-$\delta^{18}O$ space of NO_3^-. *Atmos Environ* 2021; **253**: 118387.

71. Zhu YC, Zhou SQ and Li HW *et al*. Formation pathways and sources of size-segregated nitrate aerosols in a megacity identified by dual isotopes. *Atmos Environ* 2021; **264**: 118708.

72. Lin YC, Zhang YL and Yu MY *et al*. Formation mechanisms and source apportionments of airborne nitrate aerosols at a Himalayan-Tibetan Plateau site: Insights from nitrogen and oxygen isotopic compositions. *Environ Sci Technol* 2021; **55**: 12261-12271.

73. Dong XY, Guo QJ and Han XK *et al*. The isotopic patterns and source apportionment of nitrate and ammonium in atmospheric aerosol. *Sci Total Environ* 2021; **803**: 149559.

74. Carter TS, Joyce EE and Hastings MG. Quantifying nitrate formation pathways in the equatorial pacific atmosphere from the GEOTRACES Peru-Tahiti Transect. *ACS Earth Space Chem* 2021; **5**: 2638-2651.

Text S3. Publications of simultaneous observations of HNO_3, p-NO_3^-, and NO_2 concentrations in the atmosphere.

1. Elliott EM, Kendall C and Boyer EW *et al*. Dual nitrate isotopes in dry deposition: Utility for partitioning NO_x source contributions to landscape nitrogen deposition. *J Geophys Res-Biogeo* 2009; **114**: G04020.

2. Freyer H. Seasonal variation of $^{15}N/^{14}N$ ratios in atmospheric nitrate species. *Tellus B* 1991; **43**: 30–44.

3. Freyer H, Kley D and Volz-Thomas A *et al*. On the interaction of isotopic exchange processes with photochemical reactions in atmospheric oxides of nitrogen. *J Geophys Res-Atmos* 1993; **98**: 14791–14796.

4. Nelson DM, Tsunogai U and Dong D *et al*. Triple oxygen isotopes indicate urbanization affects sources of nitrate in wet and dry atmospheric deposition. *Atmos Chem Phys* 2018; **18**: 6381–6392.

5. Savard MM, Cole A and Smirnoff A *et al*. $\delta^{15}N$ values of atmospheric N species simultaneously collected using sector-based samplers distant from sources-isotopic inheritance and fractionation. *Atmos Environ* 2017; **162**: 11–22.

6. Wojtal PK, Miller DJ and O’Conner M *et al*. Automated, high-resolution mobile collection system for the nitrogen isotopic analysis of NO_x. *J Vis Exp* 2016; **118**: e54962.

Text S4. Data sources of observations of HNO_3, p-NO_3^-, and NO_2 concentrations in the ambient atmosphere.

1. https://emep.int/publ/common_publications.html#2019

2. https://monitoring.eanet.asia/document/public/index

3. https://www3.epa.gov/ttn/amtic/supersites.html

Text S5. Publications of observations of ambient $f_{NO2/NOx}$ values.

1. Nuterman R, Mahura A and Baklanov A *et al*. Downscaling system for modeling of atmospheric composition on regional, urban and street scales. *Atmos Chem Phys* 2021; **21**: 11099–11112.

2. Hilker N, Jeong CH and Wang JM *et al*. Elucidating long-term trends, seasonal variability, and local impacts from thirteen years of near-road particle size data (2006–2019). *Sci Total Environ* 2021; **774**: 145028.

3. Cristofanelli P, Gutiérrez I and Adame JA *et al*. Interannual and seasonal variability of NO_x observed at the Mt. Cimone GAW/WMO global station (2165 mas.l., Italy). *Atmos Environ* 2021; **249**: 118245.

4. Andersen ST, Carpenter LJ and Nelson BS *et al*. Long-term NO_x measurements in the remote marine tropical troposphere. *Atmos Meas Tech* 2021; **14**: 3071–3085.

5. Zhao SM, Hu B and Gao WK *et al*. Effect of the "coal to gas" project on atmospheric NO_x during the heating period at a suburban site between Beijing and Tianjin. *Atmos Res* 2020; **241**: 104977.

6. Jurado X, Reiminger N and Vazquez J *et al*. Assessment of mean annual NO_2 concentration based on a partial dataset. *Atmos Environ* 2020; **221**: 117087.

7. Wang LJ, Wang J and Tan XD *et al*. Analysis of NO_x pollution characteristics in the atmospheric environment in Changchun city. *Atmosphere* 2020; **11**: 30.

8. Masiwal R, Sharma C and Shukla DK *et al*. Photochemistry of ozone over urban area: a case study for Delhi City. *Indian J Phys* 2019; **93**: 415–425.

9. Hůnová I, Bäumelet V and Modlík M. Long-term trends in nitrogen oxides at different types of monitoring stations in the Czech Republic. *Sci Total Environ* 2020; **699**: 134378.

10. He H, Vinnikov KY and Krotkov N *et al*. Chemical climatology of atmospheric pollutants in the eastern United States: Seasonal/diurnal cycles and contrast under clear/cloudy conditions for remote sensing. *Atmos Environ* 2019; **206**: 85–107.

11. Davis ZYW, Baray S and Mclinden CA *et al*. Estimation of NO_x and SO_2 emissions from Sarnia, Ontario, using a mobile MAX-DOAS (Multi-AXis Differential Optical Absorption Spectroscopy) and a NO_x analyzer. *Atmos Chem Phys* 2019; **19**: 13871–13889.

12. Cropper PM, Bhardwaj N and Overson DK *et al*. Source apportionment analysis of winter 2016 Neil Armstrong Academy data (West Valley City, Utah). *Atmos Environ* 2019; **219**: 116971.

13. Kasparoglu S, Incecik S and Topcu S. Spatial and temporal variation of O_3, NO and NO_2 concentrations at rural and urban sites in Marmara Region of Turkey. *Atmos Pollut Res* 2018; **9**: 1009–1020.

14. Richmond-Bryant J, Owen RC and Graham S *et al.* Estimation of on-road NO_2 concentrations, NO_2/NO_x ratios, and related roadway gradients from near-road monitoring data. *Air Qual Atmos Health* 2017; **10**: 611–625.

15. Tong SR, Hou s AND Zhang Y *et al*. Exploring the nitrous acid (HONO) formation mechanism in winter Beijing: direct emissions and heterogeneous production in urban and suburban areas. *Faraday Discussions* 2016; **189**: 213.

16. Kim DS, Jeong J and Ahn J. Characteristics in atmospheric chemistry between NO, NO_2 and O_3 at an urban site during MAPS (Megacity Air Pollution Study)-Seoul, Korea. J. Korean Society. *Atmos Environ* 2016; **32**: 422–434.

17. Hou SQ, Tong SG and Ge MF *et al*. Comparison of atmospheric nitrous acid during severe haze and clean periods in Beijing, China. *Atmos Environ* 2016; **124**: 199–206.

18. Cai CX, Kulkarni S and Zhao Z *et al*. Simulating reactive nitrogen, carbon monoxide, and ozone in California during ARCTAS-CARB 2008 with high wildfire activity. *Atmos Environ* 2016; **128**: 28–44.

19. Tiwari S, Dahiya A and Kumar N. Investigation into relationships among NO, NO_2, NO_x, O_3, and CO at an urban background site in Delhi, India. *Atmos Res* 2015; **157**: 119–126.

20. Shaw MD, Lee JD and Davison B *et al*. Airborne determination of the temporo-spatial distribution of benzene, toluene, nitrogen oxides and ozone in the boundary layer across Greater London, UK. *Atmos Chem Phys* 2015; **15**: 5083–5097.

21. Grundstrom M, Hak C and Hallquist M *et al.* Variation and co-variation of PM_{10}, particle number concentration, NO_x and NO_2 in the urban air e Relationships with wind speed, vertical temperature gradient and weather type. *Atmos Environ* 2015; **120**: 317–327.

22. Takekawa H, Chatani S and Ito A. A new approach for estimation of the effect of NO_x emission reduction on roadside NO_2 concentration in Tokyo. *Atmos Environ* 2013; **68**: 92–102.

23. Kimbrough S, Baldauf RW and Hagler GSW *et al*. Long-term continuous measurement of near-road air pollution in Las Vegas: seasonal variability in traffic emissions impact on local air quality. *Air Qual Atmos Health* 2013; **6**: 295–305.

24. Notario A, Bravo I and Adame JA *et al*. Analysis of NO, NO_2, NO_x, O_3 and oxidant (OX= O_3+NO_2) levels measured in a metropolitan area in the southwest of Iberian Peninsula. *Atmos Res* 2012; **105**: 217–226.

25. Mavroidis I and Ilia M. Trends of NO_x, NO_2 and O_3 concentrations at three different types of air quality monitoring stations in Athens, Greece. Atmos Environ 2012; **63**: 135–147.

26. Ding HY, Zhang XL and Pu WW *et al*. Change trends and corresponding meteorological character of air pollition in Beijing. *Adv Mater Res* 2011; **183**: 1209–1214.

27. Lin WL, Xu X and Ge BZ *et al*. Characteristics of gaseous pollutants at Gucheng, a rural site southwest of Beijing. *J Geophys Res* 2009; **114**: D00G14.

28. Zhang Q, Streets DG and He KB *et al*. NO_x emission trends for China, 1995–2004: The view from the ground and the view from space. *J Geophys Res* 2007; **112**: D22306.

29. Coppalle A, Delmas V and Bobbia M. Variability of NO_x and NO_2 concentrations observed at pedestrian level in the city centre of a medium sized urban area. *Atmos Environ* 2001; **35**: 5361–5369.

30. Clapp LJ and Jenkin ME. Analysis of the relationship between ambient levels of O_3, NO_2 and NO as a function of NO_x in the UK. Atmos Environ 2001; **35**: 6391–6405.

Text S6. Publications of ambient $\delta^{15}N_{NOx}$ observations.

1. Albertin S, Savarino J and Bekki S *et al*. Measurement report: Nitrogen isotopes ($\delta^{15}N$) and first quantification of oxygen isotope anomalies ($\Delta^{17}O$, $\delta^{18}O$) in atmospheric nitrogen dioxide. *Atmos Chem Phys* 2021; **21**: 10477–10497.

2. Coughlin JG, Elliott EM and Rose LA *et al.* Quantifying atmospheric reactive nitrogen concentrations, dry deposition, and isotope dynamics surrounding a Marcellus Shale well pad. *Atmos Environ* 2020; **223**: 117196.

3. Park KS, Kim H and Yu SM *et al*. Characterization of contribution of vehicle emissions to ambient NO_2 using stable isotopes. *Anal Sci Technol* 2019; **1**: 17–23.

4. Kawashima H. Seasonal trends of the stable nitrogen isotope ratio in particulate nitrogen compounds and their gaseous precursors in Akita, Japan. *Tellus B* 2019; **71**: 1–12.

5. Walters WW, Fang H and Michalski G. Summertime diurnal variations in the isotopic composition of atmospheric nitrogen dioxide at a small midwestern United States city. *Atmos Environ* 2018; **179**: 1–11.

6. Savard MM, Cole A and Smirnoff A *et al*. $\delta^{15}N$ values of atmospheric N species simultaneously collected using sector-based samplers distant from sources e Isotopic inheritance and fractionation. *Atmos Environ* 2017; **162**: 11–22.

7. Miller DJ, Wojtal PK and Clark SC *et al*. Vehicle NO_x emission plume isotopic signatures: Spatial variability across the eastern United States. *J Geophys Res-Atmos* 2017; **122**: 4698–4717.

8. Wojtal PK, Miller DJ and O'Conner M *et al*. Automated, high-resolution mobile collection system for the nitrogen isotopic analysis of NO_x. *J Vis Exp* 2016; **118**: e54962.

9. Dahal B and Hastings MG. Technical considerations for the use of passive samplers to quantify the isotopic composition of NO_x and NO_2 using the denitrifier method. Atmos Environ 2016; **143**: 60–66.

10. Buzek F, Cejkova B and Hellebrandova L *et al*. Isotope composition of NH_3, NO_x and SO_2 air pollution in the Moravia-Silesian region, Czech Republic. *Atmos Pullt Res* 2017; **8**: 221-232.

11. Savard MM, Vet R and Smirnoff A *et al*. Triple isotopic ratios to characterize atmospheric N compounds in Alberta- Work in progress. *Procedia Earth and Planetary Science* 2015; **13**: 316–319.

12. Fibiger DL, Hastings MG and Lew AF *et al*. Collection of NO and NO_2 for Isotopic Analysis of NO_x Emissions. *Anal Chem* 2016; **86**: 12115–12121.

13. Redling K, Elliott E and Bain D *et al*. Highway contributions to reactive nitrogen deposition: tracing the fate of vehicular NO_x using stable isotopes and plant biomonitors. *Biogeochemistry* 2013; **116**: 261–274.

14. Smirnoff A, Savard MM and Vet R *et al*. Nitrogen and triple oxygen isotopes in near-road air samples using chemical conversion and thermal decomposition. *Rapid Commun Mass Sp* 2012; **26**: 2791–2804.

Text S7. Publications of simultaneous $\delta^{15}N$ observations of HNO_3, p-NO_3^-, and w-NO_3^- in the atmosphere.

1. Freyer H. Seasonal variation of $^{15}N/^{14}N$ ratios in atmospheric nitrate species. *Tellus B* 1991; **43**: 30–44.

2. Elliott EM, Kendall C and Boyer EW *et al*. Dual nitrate isotopes in dry deposition: Utility for partitioning NO_x source contributions to landscape nitrogen deposition. J Geophys Res-Biogeo 2009; **114**: G04020.

3. Nelson DM, Tsunogai U and Dong D *et al*. Triple oxygen isotopes indicate urbanization affects sources of nitrate in wet and dry atmospheric deposition. *Atmos Chem Phys* 2018; **18**: 6381–6392.

4. Savard MM, Cole A and Smirnoff A *et al*. $\delta^{15}N$ values of atmospheric N species simultaneously collected using sector-based samplers distant from sources–Isotopic inheritance and fractionation. *Atmos Environ* 2017; **162**: 11–22.

Text S8. Publications of ambient $\delta^{15}N_{HNO3}$ observations.

1. Bell MD. Using stable isotopes of nitrogen and oxygen as environmental indicators of nitrogen deposition in the Sonoran Desert. PhD Thesis, University of California. 2012.

2. Elliott EM, Kendall C and Boyer EW *et al*. Dual nitrate isotopes in dry deposition: Utility for partitioning NO_x source contributions to landscape nitrogen deposition. J Geophys Res-Biogeo 2009; **114**: G04020.

3. Felix JD and Elliott EM. Isotopic composition of passively collected nitrogen dioxide emissions: vehicle, soil and livestock source signatures. *Atmos Environ* 2014; **92**: 359–366.

4. Freyer HD. Seasonal variation of $^{15}N/^{14}N$ ratios in atmospheric nitrate species. *Tellus B* 1991; **43**: 30–40 (1991).

5. Kawashima H. Formation mechanism and source apportionment of ammonium and nitrate ions in aerosol using nitrogen isotopes. *Earozoru Kenkyu* 2014; **29**: 110–116. (In Japanese with English abstract)

6. Nelson DM, Tsunogai U and Dong D *et al*. Triple oxygen isotopes indicate urbanization affects sources of nitrate in wet and dry atmospheric deposition. *Atmos Chem Phys* 2018; **18**: 6381–6392.

7. Redling K, Elliott E and Bain D *et al*. Highway contributions to reactive nitrogen deposition: tracing the fate of vehicular NO_x using stable isotopes and plant biomonitors. *Biogeochemistry* 2013; **116**: 261–274.

8. Savard MM, Cole A and Smirnoff A *et al*. $\delta^{15}N$ values of atmospheric N species simultaneously collected using sector-based samplers distant from sources–Isotopic inheritance and fractionation. *Atmos Environ* 2017; **162**: 11–22.

9. Ti CP, Wang X and Yan XY. Determining $\delta^{15}N$-NO_3^- values in soil, water, and air samples by chemical methods. *Environ Monit Assess* 2018; **190**: 341.
